# Arginine-mediated gut microbiome remodeling promotes host pulmonary immune defense against nontuberculous mycobacterial infection

**DOI:** 10.1080/19490976.2022.2073132

**Published:** 2022-05-17

**Authors:** Young Jae Kim, June-Young Lee, Jae Jin Lee, Sang Min Jeon, Prashanta Silwal, In Soo Kim, Hyeon Ji Kim, Cho Rong Park, Chaeuk Chung, Jeong Eun Han, Jee-Won Choi, Euon Jung Tak, Ji-Ho Yoo, Su-Won Jeong, Do-Yeon Kim, Warisa Ketphan, Su-Young Kim, Byung Woo Jhun, Jake Whang, Jin-Man Kim, Hyungjin Eoh, Jin-Woo Bae, Eun-Kyeong Jo

**Affiliations:** a Department of Microbiology, Chungnam National University School of Medicine Daejeon, Korea; b Infection Control Convergence Research Center, Chungnam National University School of Medicine Daejeon, Korea; c Department of Medical Science, Chungnam National University School of Medicine Daejeon, Korea; d Brain Korea 21 FOUR Project for Medical Science, Chungnam National University School of Medicine Daejeon, Korea; e Department of Life and Nanopharmaceutical Sciences and Department of Biology, Kyung Hee University, Seoul, Korea; f Department of Biomedical and Pharmaceutical Sciences, Kyung Hee University, Seoul, Korea; g Department of Molecular Microbiology and Immunology, Keck School of Medicine, University of Southern California CA, USA; h Division of Pulmonary and Critical Care, Department of Internal Medicine, Chungnam National University School of Medicine Daejeon, Korea; i Division of Pulmonary and Critical Care Medicine, Department of Medicine, Samsung Medical Center, Sungkyunkwan University School of Medicine Seoul, South Korea; j Korea Mycobacterium Resource Center (KMRC) & Basic Research Section, The Korean Institute of Tuberculosis (KIT), Cheongju, Korea; k Department of Pathology, Chungnam National University School of Medicine, Daejeon, Korea

**Keywords:** Nontuberculous mycobacteria, l-arginine, host defense, gut microbiota

## Abstract

Nontuberculous mycobacterial pulmonary diseases (NTM-PDs) are emerging as global health threats with issues of antibiotic resistance. Accumulating evidence suggests that the gut–lung axis may provide novel candidates for host-directed therapeutics against various infectious diseases. However, little is known about the gut–lung axis in the context of host protective immunity to identify new therapeutics for NTM-PDs. This study was performed to identify gut microbes and metabolites capable of conferring pulmonary immunity to NTM-PDs. Using metabolomics analysis of sera from NTM-PD patients and mouse models, we showed that the levels of l-arginine were decreased in sera from NTM-PD patients and NTM-infected mice. Oral administration of l-arginine significantly enhanced pulmonary antimicrobial activities with the expansion of IFN-γ-producing effector T cells and a shift to microbicidal (M1) macrophages in the lungs of NTM-PD model mice. Mice that received fecal microbiota transplants from l-arginine-treated mice showed increased protective host defense in the lungs against NTM-PD, whereas l-arginine-induced pulmonary host defense was attenuated in mice treated with antibiotics. Using 16S rRNA sequencing, we further showed that l-arginine administration resulted in enrichment of the gut microbiota composition with *Bifidobacterium* species. Notably, oral treatment with either *Bifidobacterium pseudolongum* or inosine enhanced antimicrobial pulmonary immune defense against NTM infection, even with multidrug-resistant clinical NTM strains. Our findings indicate that l-arginine-induced gut microbiota remodeling with enrichment of *B. pseudolongum* boosts pulmonary immune defense against NTM infection by driving the protective gut–lung axis *in vivo*.

## Introduction

The incidence, prevalence, and mortality rates of nontuberculous mycobacterial (NTM) infections are increasing globally.^1–3^ NTM pulmonary diseases (NTM-PDs) are the most common clinical manifestations of NTM infections, often caused by the slowly growing *Mycobacterium avium* (Mav) complex and the rapidly growing *Mycobacterium abscessus* complex, including *M. abscessus* subsp. *abscessus* (Mabc), *M. abscessus* subsp. *massiliense* (Mmass), and *M. abscessus* subsp. *bolletii*.^2,3^ Notably, *M. abscessus* complex is emerging as a significant cause of pulmonary infections in both immunocompetent and immunocompromised patients.^4,5^ Generally, the duration of treatment for NTM-PD is 12 months or more, often with poor outcomes. Recent meta-analyses of antibiotic therapy in NTM-PDs have shown that aminoglycoside- and macrolide-containing regimens have success rates <70%.^6,7^ In addition, the treatment is complicated and intensive due to natural and acquired antibiotic resistance.^1,8,9^ Moreover, recent studies have suggested the possible direct transmission of emergent NTM within the population,^10,11^ necessitating the development of new protective and therapeutic modalities against emerging NTM-PDs.

There is accumulating evidence that the gut microbiota and their metabolites orchestrate the immune response in human health and various diseases, including infections.^12,13^ Notably, the susceptibility and progression of NTM-PDs are associated with specific body morphotypes, such as a low body mass index (BMI), abdominal fat volume, and albumin level.^14,15^ These findings suggest that an imbalance of nutrients and metabolites may be linked to the pathogenesis of diseases associated with NTM.^14,15^ However, the roles of the gut microbiome and metabolites in the host–pathogen interaction in NTM diseases are largely unknown compared to those in tuberculosis (TB), a more intensively studied infectious disease. Advances in our understanding of gut microbiota–host crosstalk have revealed how microbial composition affects pathological responses in extraintestinal organs.^16–19^ The intestinal microbiota, which is influenced by diet, generates metabolites and bioreactive signals that mediate communication between the gut and other distant organs to maintain vital functions and to control immune responses in the body.^20,21^ However, the roles of the gut–lung axis and the mechanisms by which it modulates susceptibility and confers protection against NTM-PDs have yet to be elucidated.

l-Arginine is an essential amino acid involved in various biological processes, including elimination of nitrogenous waste products, NO synthesis, protein kinase signaling, and immune function.^22–24^ l-Arginine homeostasis is regulated by dietary uptake, *de novo* synthesis, and scavenging after protein turnover.^24^ l-Arginine deficiency, caused by reduced availability or increased catabolism, is involved in dysregulated host defenses and immunosuppression during infection and inflammation.^23,24^ Indeed, the serum concentrations of l-citrulline and l-ornithine, which are downstream metabolites of l-arginine, are lower in patients with active TB compared to healthy controls (HCs).^25–27^ Clinical trials have shown that l-arginine supplementation or ingestion of an l-arginine-rich diet can improve clinical outcomes in active TB patients,^28,29^ although other studies have found no beneficial effects.^28,30^ Notably, l-arginine supplementation improves TB symptoms and weight gain and contributes to host defense against TB, probably by increasing NO production.^22,29,31^ Although the effects of l-arginine are much less well understood in the context of NTM infections, l-arginine supplementation may serve as host-directed therapy to treat both NTM-PD and active TB. However, it is not fully understood how l-arginine in the diet exhibits beneficial effects against mycobacterial infections *in vivo*.

In this study, we first investigated the metabolic profiles of NTM-PD patients to identify metabolites that may confer protective immunity. We found that the serum l-arginine level was significantly lower in NTM-PD patients than HCs. Therefore, we established a murine model of NTM-PD infection and found that the serum l-arginine level was considerably lower following NTM pulmonary infection in these mice. Importantly, we found that the composition of the gut microbiota in mice treated with l-arginine shifted toward *Bifidobacterium* sp. enrichment. We further demonstrated the role of the gut microbiota in host protection against NTM-PDs by performing fecal microbiota transplantation (FMT) and microbiota depletion by antibiotic administration in mice. Finally, oral administration of either *Bifidobacterium pseudolongum* or the metabolite, inosine, promoted host defense against pulmonary infection caused by NTMs, even with multidrug-resistant clinical strains, by triggering the expansion of interferon (IFN)-γ-producing effector T cells and a shift toward microbicidal (M1) macrophage responses.

## Results

### *The serum of NTM-PD patients is deficient in* *l**-arginine*

The peripheral blood mononuclear cells (PBMCs) of NTM patients infected with rapidly growing Mabc and Mmass produce markedly increased levels of proinflammatory cytokines and chemokines compared to HCs.^32^ To investigate the relationship between host immunometabolic dysregulation and NTM infection outcomes, serum samples from NTM-PD patients and HCs were subjected to metabolomic analysis. The clinical characteristics of 16 patients with Mabc infection and 16 patients with Mmass infection are shown in Table 1.

**Table 1.** Baseline Characteristics of Study Patients at Diagnosis

Characteristics	Mabc (n = 16)	Mmass (n = 16)	*P*-value
Age, year	58 (± 2)	56 (± 2)	0.534
Female	14 (88)	13 (81)	0.639
Body mass index, kg/m^2^	20.8 (± 0.6)	20.7 (± 0.5)	0.886
Never smoker	15 (94)	15 (94)	>0.999
Underlying conditions			
Bronchiectasis	15 (94)	16 (100)	0.349
Previous pulmonary tuberculosis	9 (56)	6 (38)	0.303
Chronic obstructive pulmonary disease	0 (0)	0 (0)	>0.999
Diabetes mellitus	0 (0)	0 (0)	>0.999
Sinusitis	0 (0)	1 (6)	0.349
Symptoms			
Cough	9 (56)	7 (44)	0.495
Sputum	10 (63)	9 (56)	0.729
Hemoptysis	5 (31)	9 (56)	0.164
Type of disease			0.639
Noncavitary nodular bronchiectatic	13 (81)	14 (88)	
Cavitary nodular bronchiectatic	3 (19)	2 (13)	
Fibrocavitary	0 (0)	0 (0)	
Erythrocyte sedimentation rate, mm/h	33 (± 7)	35 (± 7)	0.836
C-reactive protein, mg/dl	0.21 (± 0.09)	0.27 (± 0.08)	0.641
Albumin, g/dl	4.3 (± 0.1)	4.3 (± 0.10)	0.966
Sputum smear positivity	3 (19)	3 (19)	>0.999

Data are presented as means (± standard error of the mean) or numbers (%). Mabc = *M. abscessus* subsp. *abscessus*, Mmass = *M. abscessus* subsp. *massiliense*.

**Figure 1.**
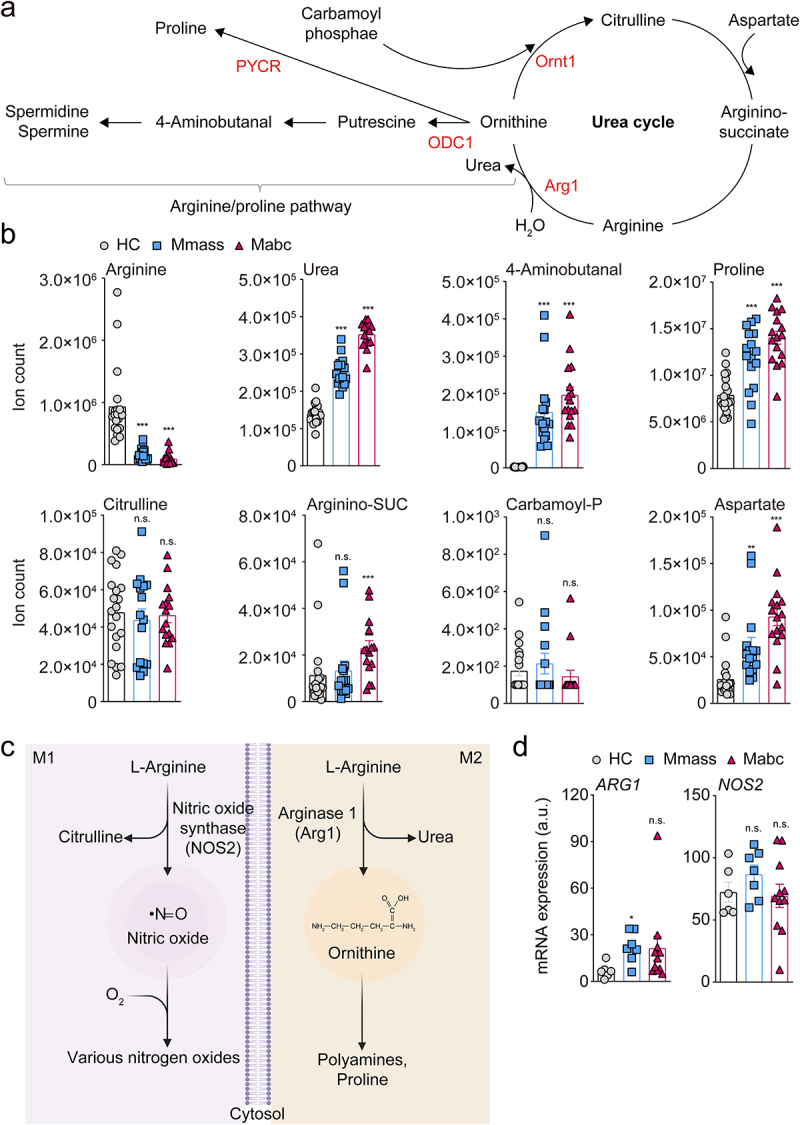
L-Arginine degradation pathways are biased in NTM-PD patients.

Host metabolic dysregulation in NTM-PD patients was determined by comparing the relative abundances of ~210 metabolites using the Metlin database (http://metlin.scripps.edu). Using MetaboAnalyst (ver. 5.0), we identified a subset of metabolites uniquely altered by NTM infection via multivariate hierarchical clustering (Figure S1a). Then we performed principal component analysis of the metabolites with significantly different profiles, which indicated that the host metabolism of Mabc/Mmass-infected individuals was metabolically different from that of HCs (Figure S1b). Fold change analysis indicated significantly higher serum levels of 124 metabolites and lower levels of 23 metabolites in NTM patients compared to HCs (Figure S1c and Table S1). The serum levels of l-arginine were significantly lower in NTM patients (Figure S1c). Pathway enrichment analysis revealed mechanistic insight of foregoing metabolite change reflecting significant associations between the reduced l-arginine level and various pathways, particularly the urea cycle and arginine/proline metabolism, in NTM-PD patients (Figure S1d). In addition, targeted metabolomics analysis showed that NTM patients had elevated levels of tryptophan and reduced levels of l-arginine and glutamine; these amino acids have immunoregulatory functions in macrophages (Table S1). l-arginine is metabolized via the arginine/proline pathway and the urea cycle (Figure 1a). Targeted metabolomics analysis showed that serum l-arginine levels were significantly lower in NTM-PD patients than HCs (Figure 1b). This analysis also showed reciprocal accumulation of urea and arginine/proline pathway intermediates, such as 4-aminobutanal and proline, in NTM-PD patients (Figure 1b). These observations suggest that l-arginine is preferentially metabolized for the biosynthesis of polyamine pathway intermediates as opposed to the biosynthesis of NO biosynthetic pathway intermediates (Figure 1c).^33,34^ Furthermore, we found no significant differences in the concentration of citrulline, a precursor of l-arginine, between HCs and NTM-PD patients, although a minor but significant increase in arginino-succinate was detected in patients with Mabc infection compared to HCs (Figure 1b). These data suggest that l-arginine depletion is a result of rapid consumption rather than reduced biosynthesis. Intriguingly, we also found that the abundance of carbamoyl phosphate in NTM-PD patients was similar to that in HCs but the abundance of aspartate was much higher in NTM-PD patients (Figure 1b). Changes in TCA cycle activity may be responsible for remodeling of the catalytic activities involved in consuming l-arginine in NTM-PD patients. Targeted metabolomics analysis suggested that NTM infection may modulate the host immune system by preventing the metabolic shift toward the M1-like macrophage state (Figure 1c).

(**a**) Schematic representation of the arginine metabolism pathway. Arginine catalysis can be divided into two pathways: the nitric oxide (NO)-citrulline cycle (seen in M1 macrophages) and ornithine-polyamine/proline pathway (seen in M2 macrophages). Enzymes are shown in red. Arg1, Arginase 1; ODC1, ornithine decarboxylase; Ornt1, ornithine translocase; PYCR, pyrroline-5-carboxylate reductase. (**b**) Intermediates of the arginine biosynthesis metabolism of sera from HCs (gray circles; *n* = 20), Mmass-infected patients (blue squares; *n* = 16), and Mabc-infected patients (red triangles; *n* = 16). Total bar height indicates the pool size ion counts of the intermediates. (**c**) M1 macrophages use the NO-citrulline cycle to enhance the antimicrobial activity by releasing proinflammatory cytokines, NO, or reactive oxygen species. M2 macrophages induce Arg1 to enhance the biosynthesis of ornithine, polyamine, and proline. The figure was created with BioRender. (**a**) *ARG1* and *NOS2* mRNA expression levels of PBMCs from HCs (gray circles; *n* = 6), Mmass-infected patients (blue squares; *n* = 7), and Mabc-infected patients (red triangles; *n* = 11) using nCounter assay. Statistical significance was calculated by one-way ANOVA with Tukey’s multiple comparison test (**b**) and Kruskal–Wallis test with Dunn’s multiple comparison test (**d**). Error bars denote ± SEM. *P < 0.05; **P < 0.01; ***P < 0.001. Arginino-SUC, arginino-succinate; a.u., arbitrary units; Carbamoyl-P, carbamoyl phosphate; n.s., not significant.

Macrophages have two l-arginine degradation pathways catalyzed by inducible nitric oxide synthase (iNOS) and arginase 1 (Arg1) in M1 and M2 macrophages, respectively (Figure 1c, d). To determine which pathways were activated during NTM infection, we reexamined our previous transcriptomics data comparing the mRNA expression profiles of PBMCs isolated from NTM-PD patients compared to HCs,^32^ and analyzed the levels of *ARG1* and iNOS *(NOS2)* expression. *ARG1* level was upregulated in the PBMCs of patients infected with Mmass compared to the HCs, while there were no significant differences in iNOS pathway regulation (Figure 1d), supporting the metabolomics data from the present study. These data strongly suggest that l-arginine level is significantly reduced in NTM-PD patients.

### *L* *-Arginine administration reduces pulmonary bacterial load in NTM-infected mice*

**Figure 2.**
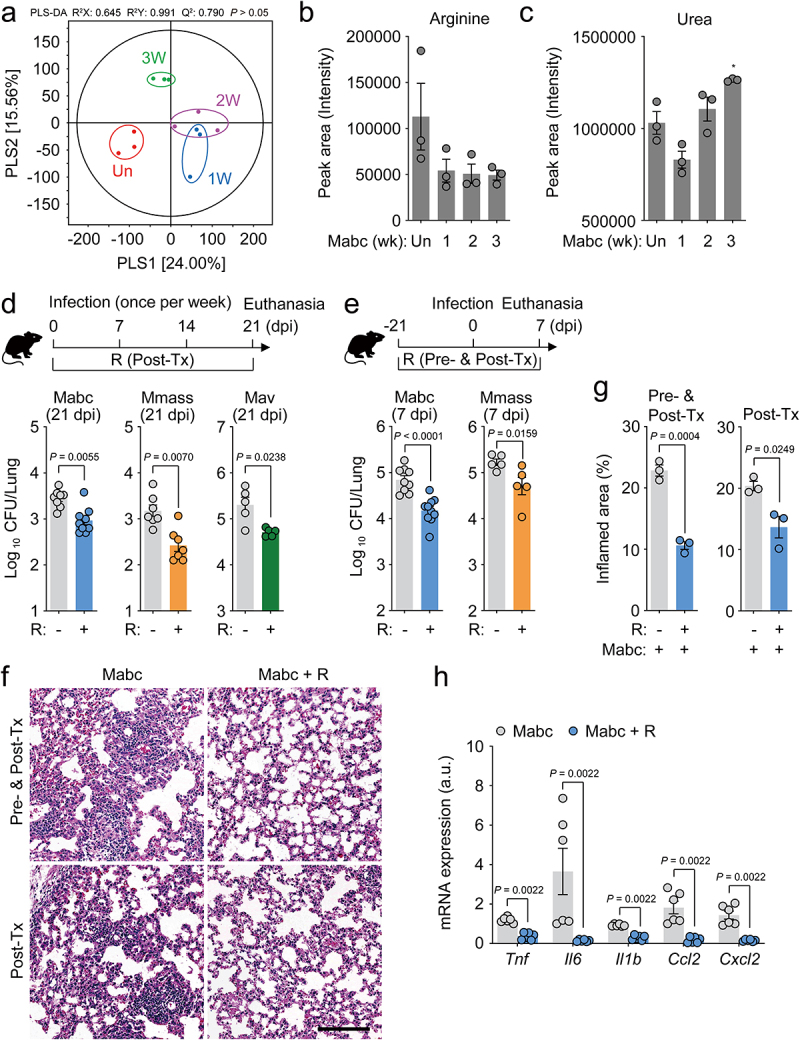
L-Arginine administration attenuated lung bacterial load during NTM-PD.

Next, we investigated whether infection of mice with NTMs mimicked the metabolic profiles of NTM-PD patients. For this purpose, we established an NTM-PD mouse model by intranasal infection with Mabc. Partial least squares discriminant analysis was performed to visualize the clustering of samples (Figure 2a), and the results showed that Mabc infection affected the metabolomic profile of the mice. Based on these results, we selected significantly different metabolites between infected and uninfected control mice using variable importance in projection (VIP) value (>1.0). A total of 64 serum metabolites, including organic compounds, amino acids, carbohydrates, phosphate esters, fatty acids, and lipids, were identified, and the relative concentrations of each metabolite were calculated (Figure S2a). l-Arginine levels were clearly, but not significantly, lower in the serum of Mabc-infected mice at 7 days postinfection (dpi) compared to uninfected controls (Figure 2b). l-Arginine is catabolized mainly by Arg1 and iNOS, resulting in the production of urea, proline, glutamate, polyamines, NO, creatine, and agmatine.^35^ Metabolites involved in l-arginine metabolic networks were further analyzed. We found a significantly higher urea concentration but a substantially lower concentration of l-citrulline, the metabolite produced by iNOS, in the serum of mice intranasally infected with NTM at 21 dpi (Figure 2c, Figure S2a,b). These results indicate that l-arginine depletion is accompanied by increased urea level in the NTM-PD mouse infection model, consistent with that seen in human NTM-PD patients.

(**a**–**c**) Metabolomic analysis of sera from uninfected (Un) or Mabc-infected mice for 1 (1W), 2 (2W), and 3 weeks (3W) (*n* = 3 mice per group). (**a**) Partial least squares discriminate analysis (PLS-DA) plots. (**b** and **c**) Comparisons of the peak areas for (**b**) arginine and (**c**) urea. (**d**) Schematic diagram of Post-Tx experiment showing time points for vehicle or l-arginine administration after infection with NTM (top) and NTM loads in murine lungs (bottom). Mice were intranasally infected three times at 0, 7, and 14 days dpi with Mabc (left; *n* = 8–9 mice per group), Mmass (middle; *n* = 7 mice per group), or Mav (right; *n* = 5 mice per group), euthanized at 21 dpi, and the lungs were resected. (**e**) Schematic diagram of Pre-and Post-Tx experiment showing administration time points for vehicle or l-arginine before infection (top) and NTM bacterial loads (CFU) in the murine lungs (bottom). After 21 days of treatment with vehicle or l-arginine, mice were intranasally infected with Mabc (left; *n* = 8–9 mice per group) or Mmass (right; *n* = 5 mice per group), euthanized at 7 dpi, and the lungs were resected. (**f**) Representative histopathological images (scale bar = 300 μm) and (**g**) quantitative analysis of the inflamed area of the lung tissues from mice in Pre- and Post-Tx (**e** top) and Post-Tx (**d** top) experiments using hematoxylin and eosin staining. (**h**) Relative *Tnf, Il6, Il1b, Ccl2*, and *Cxcl2* mRNA expression in the lung tissues of mice in the Pre- and Post-Tx (**e** top) experiment by qRT-PCR analysis. Statistical significance was calculated by the unpaired t test (**b** and **c**) and Mann–Whitney U test (**d**, **e**, **g**, and **h**). Data are representative of at least three independent experiments, and are presented as the mean ± SEM. *P < 0.05. a.u., arbitrary units; CFU, colony forming units; dpi, days postinfection; R, l-arginine.

The l-arginine deficiency in the sera of both NTM-infected humans and Mabc-infected mice prompted us to examine the effects of l-arginine supplementation on host defense of mice with NTM-PD. We administered l-arginine (3.75 mg/mL in drinking water) to mice infected with Mabc, Mmass, and Mav, and compared the *in vivo* colony forming units (CFU) of NTM bacteria with those in vehicle-treated NTM-infected control mice at 21 dpi. As shown in Figure 2d, oral l-arginine administration to NTM-infected mice for 3 weeks significantly interfered with the *in vivo* bacterial replication of Mabc, Mmass, and Mav, compared to vehicle-treated controls.

Next, we investigated whether pretreatment of mice with l-arginine had a protective effect against the early phase of NTM infection. Briefly, mice were pretreated with l-arginine (3.75 mg/mL in drinking water) for 3 weeks, followed by infection with Mabc or Mmass, and further treated with l-arginine for 1 week (Figure 2e, top). The results showed significantly suppressed *in vivo* Mabc and Mmass growth at 7 dpi in mice treated with l-arginine both pre- and postinfection compared to vehicle-treated controls (Figure 2e). Consistent with these findings, histological analyses of the lungs of mice infected with Mabc revealed less severe pathology and inflammation in mice treated with l-arginine compared to controls (Figure 2f,g). In a previous study, we found that PBMCs from NTM-PD patients showed significantly elevated pathological inflammation (characterized by increased tumor necrosis factor [*TNF*], interleukin [*IL*]*-6, IL-1B*, chemokine ligand 2 [*CCL2*], and C-X-C motif chemokine ligand 2 [*CXCL2*] expression) compared to HCs.^32^ Therefore, we examined the effects of l-arginine pretreatment on hyperactivation of inflammation induced by Mabc infection in mice. In the lungs of l-arginine-treated mice at 7 dpi, the expression levels of inflammatory cytokines and chemokines were markedly lower in mice treated with l-arginine after Mabc infection than in vehicle-treated controls (Figure 2h). Taken together, these observations strongly suggest that pre- and/or posttreatment with l-arginine significantly inhibited *in vivo* NTM bacterial growth and pathological inflammation in the lungs of NTM-infected mice.

### *L**-Arginine enhances M1 macrophage and protective Th1 responses* in vivo *in NTM-infected mice*

**Figure 3.**
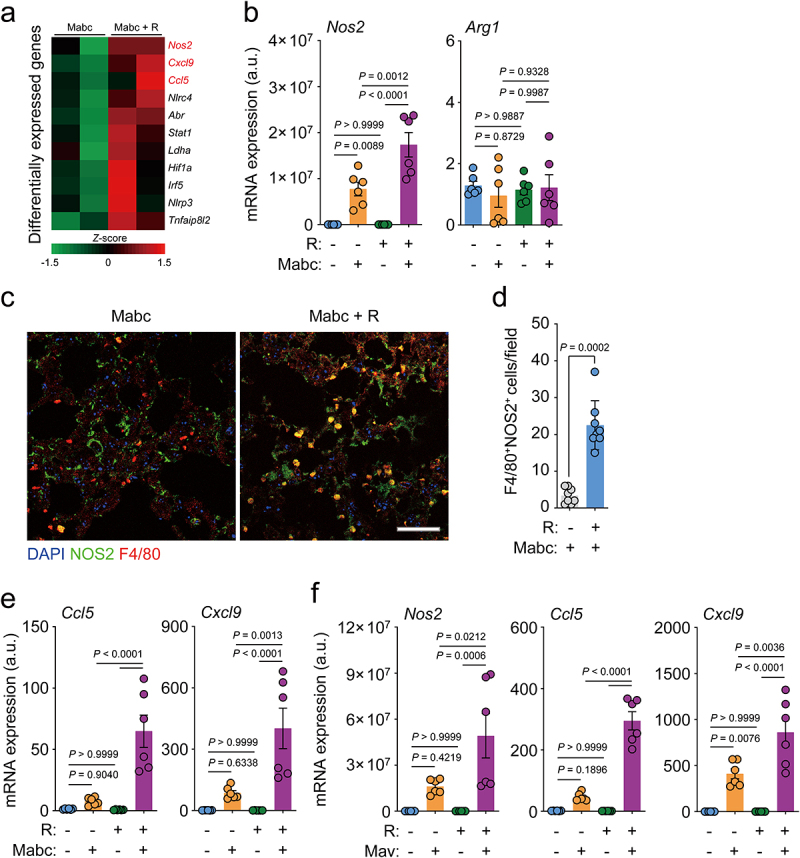
L-Arginine treatment promotes M1 shift in the lung tissues of mice with NTM-PD. For the NTM-infected group, the *in vivo* experimental schedule was the same as in Figure 2d top.

To comprehensively examine the protective effects of l-arginine administration on the lung transcriptome during NTM infection, we compared the mRNA profiles of the lungs of Mabc-infected mice treated with l-arginine (or not treated). RNA sequencing (RNA-Seq) analysis showed that several genes involved in inflammatory responses were differentially expressed in the lungs of untreated versus l-arginine-treated mice after Mabc infection (Figure 3a). By quantitative reverse transcription-polymerase chain reaction (qRT-PCR) analysis, we further validated the differential expression of candidate genes in the lung tissues from four different groups of mice, as shown in Figure 3b. Notably, the mRNA and protein levels of iNOS, but not Arg1, were significantly higher in the lung tissues from Mabc-infected/l-arginine-treated mice than in Mabc-infected mice without l-arginine treatment (Figure 3b–d; treatment schedule as shown in Figure 2d). In addition, the expression levels of *Ccl5* and *Cxcl9*, which are associated with M1 macrophage polarization,^36,37^ were also higher in the lung tissues of l-arginine-treated/Mabc-infected mice than Mabc-infected mice without l-arginine treatment (Figure 3a). We confirmed these results by qPCR analysis, which revealed that *Ccl5* and *Cxcl9* mRNA levels were markedly elevated in Mabc-infected/l-arginine-treated mice compared to untreated Mabc-infected mice without l-arginine treatment (Figure 3e). Furthermore, the mRNA levels of *iNos2, Ccl5*, and *Cxcl9* were significantly higher in Mav-infected mice treated with l-arginine than in untreated Mav-infected controls (Figure 3f). Taken together, these data suggest that l-arginine administration markedly enhances M1 macrophage responses in the lungs during NTM infection.

(**a**) Heatmap analysis of differentially expressed gene sets between vehicle- and l-arginine treated lung tissues from Mabc-infected mice. The color scale bar of the heatmap represents z-scores of normalized gene counts. (**b**) *Nos2* and *Arg1* mRNA expression levels in the lung tissues of four different groups of mice determined by qRT-PCR (*n* = 6 mice per group). (**c**) Representative immunofluorescence images (scale bar = 30 μm) and (**d**) quantitative analysis of F4/80^+^NOS2^+^ cells per field of the lung tissues from Mabc-infected mice treated with vehicle or l-arginine. (**e**) *Ccl5* and *Cxcl9* mRNA expression levels in the lung tissues of four different groups of mice determined by qRT-PCR (n = 6 mice per group). (**e**) *Nos2, Ccl5*, and *Cxcl9* mRNA expression levels in the lung tissues of four different groups of mice determined by qRT-PCR (n = 6 mice per group). Statistical significance was calculated by one-way ANOVA with Tukey’s multiple comparison test (**b**, **e**, and **f**) and Mann–Whitney U test (**d**). Data are representative of at least three independent experiments, and are presented as the mean ± SEM. a.u., arbitrary units; R, l-arginine.

**Figure 4.**
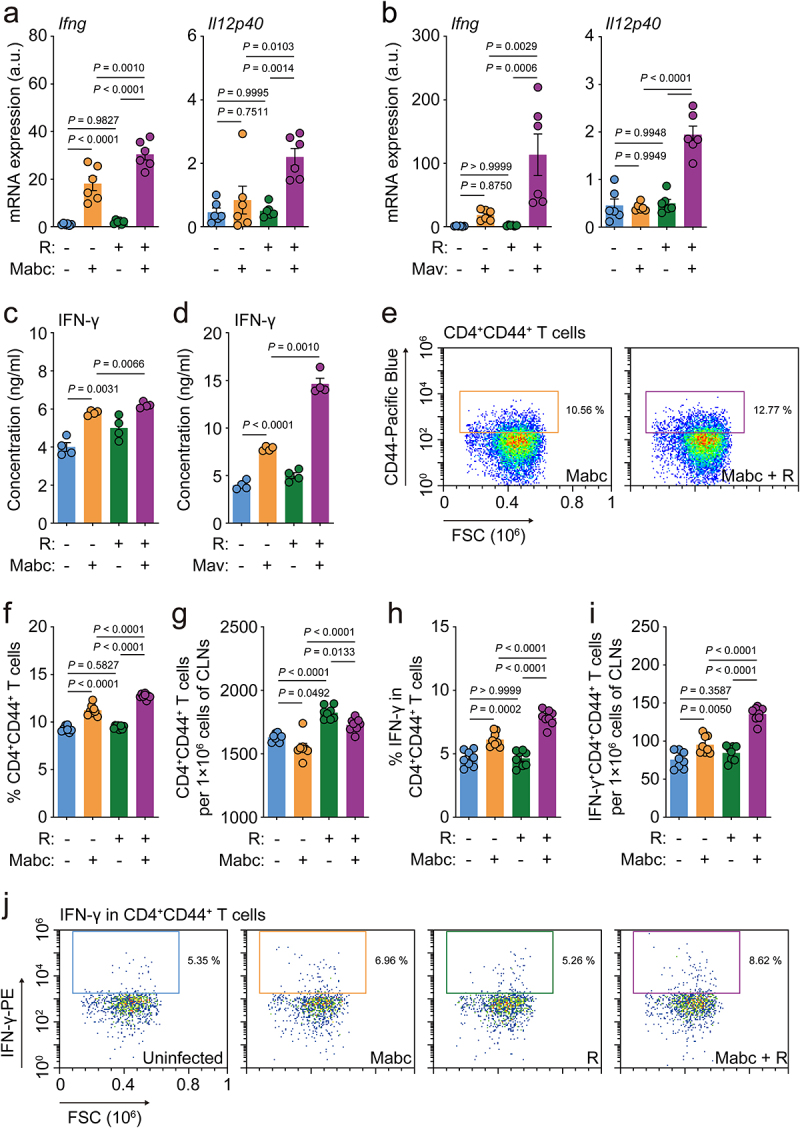
L-Arginine treatment upregulates th1 immune responses in the lung tissues of mice with NTM-PD. The *in vivo* experimental design was the same as in Figure 2d top.

Next, due to the marked upregulation of *iNOS*, we examined the expression of IFN-γ, which is mainly produced by T helper 1 (Th1) cells, and its related cytokine IL-12B (*Il12p40*). We found that the mRNA levels of IFN-γ and IL-12B were significantly higher in Mabc- and Mav-infected mice treated with l-arginine compared to corresponding untreated infected mice (Figure 4a,b). Furthermore, we found that IFN-γ secretion was considerably elevated in the lung tissues of NTM-infected/l-arginine-treated mice compared to NTM-infected mice without l-arginine treatment (Figure 4c–d, for Mabc and Mav, respectively). Moreover, the IFN-γ-producing populations of Th1 effector T cells in the cervical lymph nodes were compared between vehicle- and l-arginine-treated mice after Mabc infection. The gating strategies of flow cytometric analysis are shown in Figure S3. The CD4^+^CD44^+^ T cell population in the cervical lymph nodes of Mabc-infected mice was significantly increased in NTM-infected mice following l-arginine treatment (Figure 4e–g). It was also noted that IFN-γ-producing CD4^+^ effector T cells were significantly more abundant in the cervical lymph nodes of Mabc-infected mice treated with l-arginine than those treated with vehicle alone (Figure 4h–j). Therefore, our findings indicate that oral administration of l-arginine significantly enhances M1 macrophages and Th1 protective immune responses in the lungs of NTM-infected mice.

(**a** and **b**) *Ifng* and *Il12p40* mRNA expression levels in the lung tissues of four different groups of mice with (**a**) Mabc or (**b**) Mav infection determined by qRT-PCR (n = 8 mice per group). (**c** and **d**) IFN-γ concentration in the supernatants of lung lysates from (**c**) Mabc- or (**d**) Mav-infected mice measured with ELISA (*n* = 4 mice per group). (e–j) Flow cytometric analysis of immune cells from cervical lymph nodes of four different groups of mice (*n* = 8 mice per group). (**e**) Representative dot plots and (**f**) the proportion of CD44-positive cells in CD4^+^ T cells. (**g**) Numbers of CD4^+^CD44^+^ T cells per 1×10^6^ cells. (**h**) The proportion of IFN-γ-positive cells in CD4^+^CD44^+^ T cells. (**i**) IFN-γ^+^CD4^+^CD44^+^ T cells per 1×10^6^ cells. (**j**) Representative dot plots of IFN-γ-positive cells in CD4^+^CD44^+^ T cells. Statistical significance was calculated by one-way ANOVA with Tukey’s multiple comparison test (**a**, **b**, and **f**–**i**) and the unpaired *t* test (**c** and **d**). Data are representative of at least three independent experiments, and are presented as the mean ± SEM. a.u., arbitrary units; R, l-arginine.

### *L* *-Arginine shifts in murine gut microbiota composition and bacterial functional pathways*

**Figure 5.**
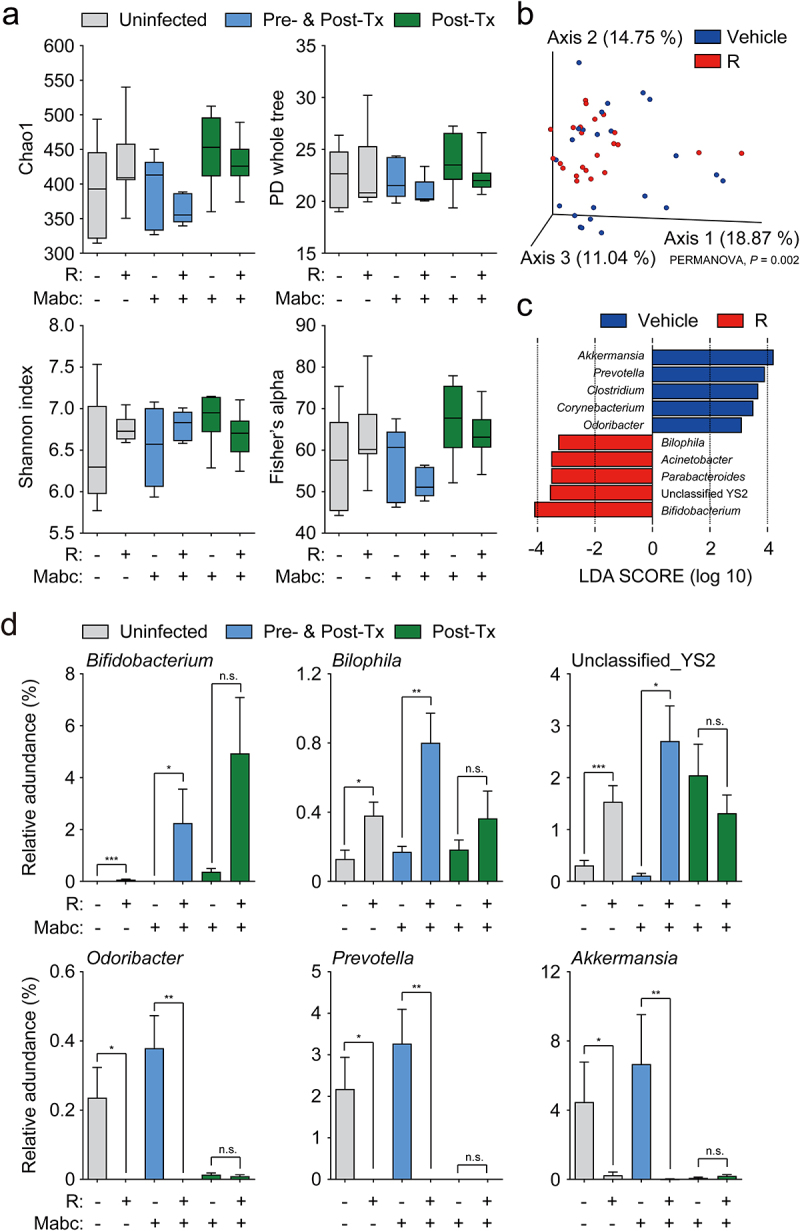
The gut microbiome of l-arginine-treated mice is enriched with *Bifidobacterium* sp. The experimental designs of Post-Tx experiments (green bar) and Pre- and Post-Tx experiments (blue bar) were the same as Figure 2(d) top and (e) top, respectively. For the Pre- and Post-Tx experiment (blue bars), feces were collected at 7 dpi (*n* = 5 mice per group). For the Post-Tx experiment (green bars), feces were collected at 21 dpi (*n* = 9 mice per group). In the uninfected control group (gray bars), mice were matched for sex, age, and weight, and maintained simultaneously with the Post-Tx experiment (*n* = 9 mice per group). The collected feces were analyzed by 16S rRNA amplicon sequencing.

The gut microbiota utilizes and generates various compounds that regulate the physiology of distal organs,^21,38,39^ and it is involved in the host health and immune homeostasis.^40–42^ l-Arginine supplementation attenuates intestinal dysbiosis in murine colitis models by increasing the levels of anticolitogenic bacteria.^43^ Furthermore, it alleviates intestinal injury in broiler chickens infected with *Clostridium perfringens* by modulating gut microbiota.^44^ To investigate the relationship between l-arginine and the gut microbiota during Mabc infection, we compared the gut microbiota compositions of uninfected and Mabc-infected mice treated with l-arginine (or not treated; experimental schedule, see Figure 2d,e). The α-diversity indices were not changed by l-arginine supplementation and Mabc infection (Figure 5a). The results of principal coordinate analysis (PCoA) based on unweighted UniFrac distances showed that six experimental groups were significantly separated by each group (PERMANOVA, *P* = 9.999e^–05^) (Figure S4a). Also, PCoA based on unweighted UniFrac distances revealed significant differences in the gut microbiota structures between vehicle- and l-arginine-treated groups (PERMANOVA, *P* = 0.002) (Figure 5b). Mabc infection also affected the gut microbiota composition, and PCoA showed a clear separation between uninfected and Mabc-infected groups (PERMANOVA, *P* = 9.999e^–05^) (Figure S4b). Furthermore, PCoA based on weighted UniFrac distances also showed significant separation following l-arginine supplementation (PERMANOVA, *P* = 0.029) and Mabc infection (PERMANOVA, *P* = 0.044) (Figure S4c,d).

(**a**) Alpha diversity between the presence and time points of Mabc infection and l-arginine treatment was analyzed using Chao1, Shannon index, Fisher’s alpha, and phylogenetic diversity (PD) whole tree metrics. (**b**) Results of principal coordinate analysis (PCoA) based on unweighted UniFrac distance metrics between mice treated with vehicle (blue) or l-arginine (red). (**c**) Significantly enriched bacterial taxa in feces from mice treated with vehicle (blue) or l-arginine (red) were identified by linear discriminant analysis (LDA) effect size (LEfSe) analysis (LDA score > 3.0, P < 0.05). (d) The relative abundances of the genera *Bifidobacterium*, *Bilophila*, *Odoribacter*, *Prevotella*, *Akkermansia*, and unclassified YS2 are shown as box plots. Statistical significance was calculated using the Mann–Whitney U test (**a** and **d**) and PERMANOVA with 999 permutations (**b**). Error bars are denoted ± SEM. *P < 0.05; **P < 0.01; ***P < 0.001. n.s., not significant; R, l-arginine.

We then compared the relative abundances of major taxa (>0.5% of the mean abundance) at the genus level (Figure S4e). The abundance of *Bifidobacterium* was increased and that of *Akkermansia* was reduced in the l-arginine-treated group. Then we performed linear discriminant analysis (LDA) effect size (LEfSe) analysis to identify differentially enriched bacterial taxa associated with l-arginine supplementation and Mabc infection (LDA score >3.0, *P* < 0.05). LEfSe analysis revealed accumulation of *Bifidobacterium, Parabacteroides, Acinetobacter, Bilophila*, and unclassified YS2 genera, but reductions in *Akkermansia, Prevotella, Clostridium, Corynebacterium*, and *Odoribacter* in the l-arginine-treated group compared to the untreated group (Figure 5c). Moreover, we found that *Ruminococcus, Bifidobacterium, Allobaculum, Turicibacter*, unclassified *Paraprevotellaceae*, and *Parabacteroides* were enriched in the Mabc-infected group; however, no differentially enriched bacteria taxa were identified in the uninfected group (Figure S4f). According to statistical analysis of the LEfSe data using *t* tests, the relative abundances of *Bifidobacterium, Bilophila*, and unclassified YS2 were significantly higher in mice treated with l-arginine compared to untreated mice (Figure 5d). By contrast, the levels of *Odoribacter, Prevotella*, and *Akkermansia* were considerably reduced following l-arginine supplementation (Figure 5d).

Next, we investigated which gut microbial functional pathways were associated with l-arginine supplementation and Mabc infection via phylogenetic investigation of communities by reconstruction of unobserved states.^45^ The predicted abundances of MetaCyc pathways were analyzed by PCoA based on binary Jaccard distances. The PCoA plots showed significant segregation between the untreated and l-arginine-treated groups (PERMANOVA, *P* = 0.045) and between the uninfected and Mabc-infected groups (PERMANOVA, *P* = 0.002) (Figure S5a,b). LEfSe analysis was applied to identify which predicted pathways were related to l-arginine supplementation and Mabc infection (LDA score >2.0, *P* < 0.05). In the l-arginine-treated group, 21 pathways were differentially enriched; we found that glucose and xylose degradation, *N*-acetylneuraminate degradation, thiazole biosynthesis, thiamine diphosphate biosynthesis, glycolysis, and the Entner–Doudoroff pathway were enriched, while menaquinol biosynthesis, heme biosynthesis from glutamate, phylloquinol biosynthesis, and fucose and rhamnose degradation were decreased (Figure S5c). In the Mabc-infected groups, 49 pathways were significantly altered, including increases in purine nucleobase degradation, l-glutamate and l-glutamine biosynthesis, adenosine nucleotide degradation, guanosine nucleotide degradation, and l-methionine, l-lysine, and l-arginine biosynthesis, and decreases in palmitate biosynthesis, oleate biosynthesis, mycolate biosynthesis, and stearate biosynthesis pathways (Figure S5d). Our findings suggest that the gut microbiota and their metabolic pathways were significantly affected by l-arginine treatment during Mabc infection.

### Fecal microbiota transplantation and microbiota dysregulation by antibiotics demonstrate the role of the gut microbiota in host defenses against NTM-PD

**Figure 6.**
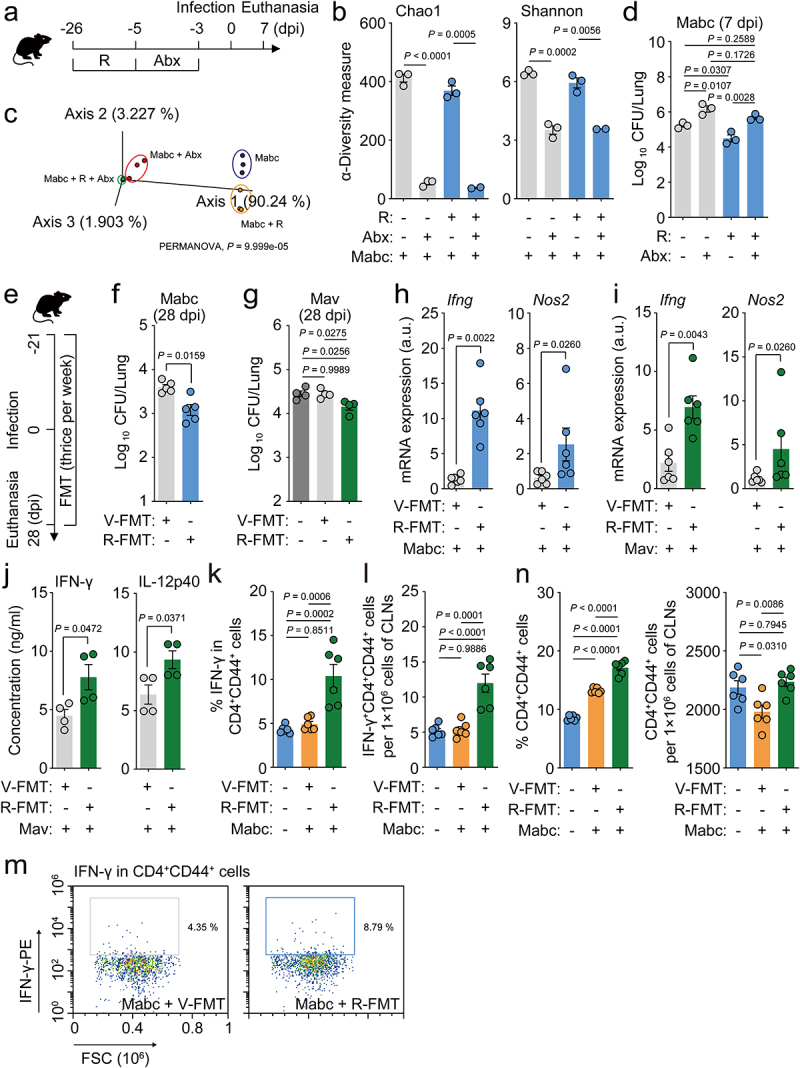
L-Arginine-mediated gut microbiome remodeling contributes to antimicrobial host defense against NTM-PD.

We found that oral treatment with l-arginine triggered antimicrobial M1 macrophage and Th1 effector responses in the lungs and significant changes in the gut microbiota composition both in the presence and absence of Mabc infection. Therefore, we examined the relationship between gut microbiota remodeling and host defense against Mabc infection. To investigate the role of the intestinal microbiota in NTM-PD, we administered l-arginine and an antibiotic cocktail (ABX) to C57BL/6 mice for 3 days as described previously,^46,47^ and then the mice were intranasally challenged with Mabc (Figure 6a). This method has been used previously to deplete the intestinal microbiota in mice.^47,48^ In parallel, control mice without ABX treatment were challenged with an equivalent concentration (in CFU) of Mabc. 16S rRNA gene sequencing analysis of the gut microbiota revealed that ABX treatment significantly reduced the bacterial diversity indices, regardless of l-arginine treatment (Figure 6b). A PCoA plot based on unweighted UniFrac distances revealed that ABX treatment induced significant changes in the gut microbial community structure (PERMANOVA, *P* = 9.999e^–05^) (Figure 6c). The Mabc bacterial loads were significantly higher in the lungs of mice treated with ABX compared to untreated mice (Figure 6d). Although pre-treatment with l-arginine resulted in reduced *in vivo* bacterial loads in the lung tissues of Mabc-infected mice, these inhibitory effects were absent in ABX-treated mice (Figure 6d), suggesting that l-arginine confers protection from pulmonary pathogens via gut microbiota remodeling in the host.

(**a**–**d**) Mice were orally administered vehicle or l-arginine for 21 days and treated with or without antibiotics for 3 days prior to intranasal infection with Mabc. Mice were euthanized at 7 dpi for collection of samples (feces and lungs for 16S rRNA amplicon sequencing and CFU assay, respectively). (**a**) Schematic diagram of the experimental time points. (**b**) Alpha diversity (Chao1 and Shannon index) and (**c**) PCoA plot based on unweighted UniFrac distance metrics from the mouse feces (n = 2–3 mice per group). (**d**) Lung bacterial loads (CFU) in four different groups of infected mice (n = 3 mice per group). (**e**–**n**) Mice were subjected to fecal microbiota transplantation (FMT) with feces from mice treated with l-arginine (R-FMT) or vehicle (V-FMT) for a total of 7 weeks three times per week. (**e**) Schematic diagram of the FMT experiments. (**f** and **g**) Lung bacterial loads (CFU) of mice infected with (**f**) Mabc (*n* = 5 mice per group) or (**g**) Mav (n = 4 mice per group). (**h** and **i**) *Ifng* and *Nos2* mRNA expression levels in the lung tissues of mice infected with (**h**) Mabc or (**i**) Mav determined by qRT-PCR. (**j**) IFN-γ and IL-12p40 concentrations in the supernatants of lung lysates from Mav-infected mice measured with ELISA (n = 4 mice per group). (**k**–**n**) Flow cytometric analysis of cervical lymph nodes from the different groups of mice (n = 6 mice per group). (**k**) The proportion of IFN-γ-positive cells in CD4^+^CD44^+^ T cells. (**l**) IFN-γ^+^CD4^+^CD44^+^ T cell numbers (per 1×10^6^ cells). (**m**) Representative dot plots of IFN-γ-positive CD4^+^CD44^+^ cells. (**n**) The proportions of CD44-positive CD4^+^ (left) and CD4^+^CD44^+^ T cell numbers (per 1×10^6^, right). Statistical significance was calculated with the unpaired *t* test (b and j), PERMANOVA with 999 permutations (c), one-way ANOVA with Tukey’s multiple comparison test (**d**, **g**, **k**, **l**, and **n**), and Mann–Whitney U test (**f**, **h**, and **i**). Data are representative of at least three independent experiments, and are presented as the mean ± SEM. Abx. antibiotics; a.u., arbitrary units; CFU, colony forming units; dpi, days postinfection; R, l-arginine.

We further investigated the relationship between the intestinal microbiota and the antimicrobial effects of l-arginine by transplanting fresh feces from l-arginine-treated C57BL/6 mice into mice before and after infection with Mabc (Figure 6e). FMT from l-arginine-treated mice resulted in decreases in Mabc and Mav bacterial loads in the lung tissues of infected mice (Figure 6f,g). In addition, the *Ifng* and *Nos2* mRNA expression levels were significantly higher in the lungs of Mabc- and Mav-infected mice that underwent FMT from l-arginine-treated mice compared to controls that received FMT from vehicle-treated mice (Figure 6 h and i, respectively). Furthermore, FMT from l-arginine-treated mice increased IFN-γ and IL-12B production in the lung tissues of Mav-infected mice compared to control mice (Figure 6j). Next, we investigated whether FMT from l-arginine-treated mice affected the number of protective IFN-γ-producing CD44^+^ T cells in the cervical lymph nodes following NTM lung infection. FMT from l-arginine-treated mice caused significant increases in IFN-γ-producing CD4^+^CD44^+^ T cell (Figure 6k–m) and CD4^+^CD44^+^ T cell populations (Figure 6n) in the cervical lymph nodes compared to control mice during Mabc infection. Taken together, these data strongly suggest that l-arginine-mediated gut microbiota remodeling contributes to pulmonary host defense against NTM infection.

### Bifidobacterium pseudolongum administration promoted protective pulmonary immune responses against NTM infection

As the genus *Bifidobacterium* was significantly enriched in l-arginine-treated mice (Figure 5c), we evaluated the relationship between the *Bifidobacterium* identified in our analyses and NTM infection. Two amplicon sequence variants belonging to the genus *Bifidobacterium* were identified. 16S rRNA gene sequencing analysis identified the sequences as *Bifidobacterium pseudolongum* and *Bifidobacterium animalis*, respectively. Therefore, these species were selected for the following experiments.

**Figure 7.**
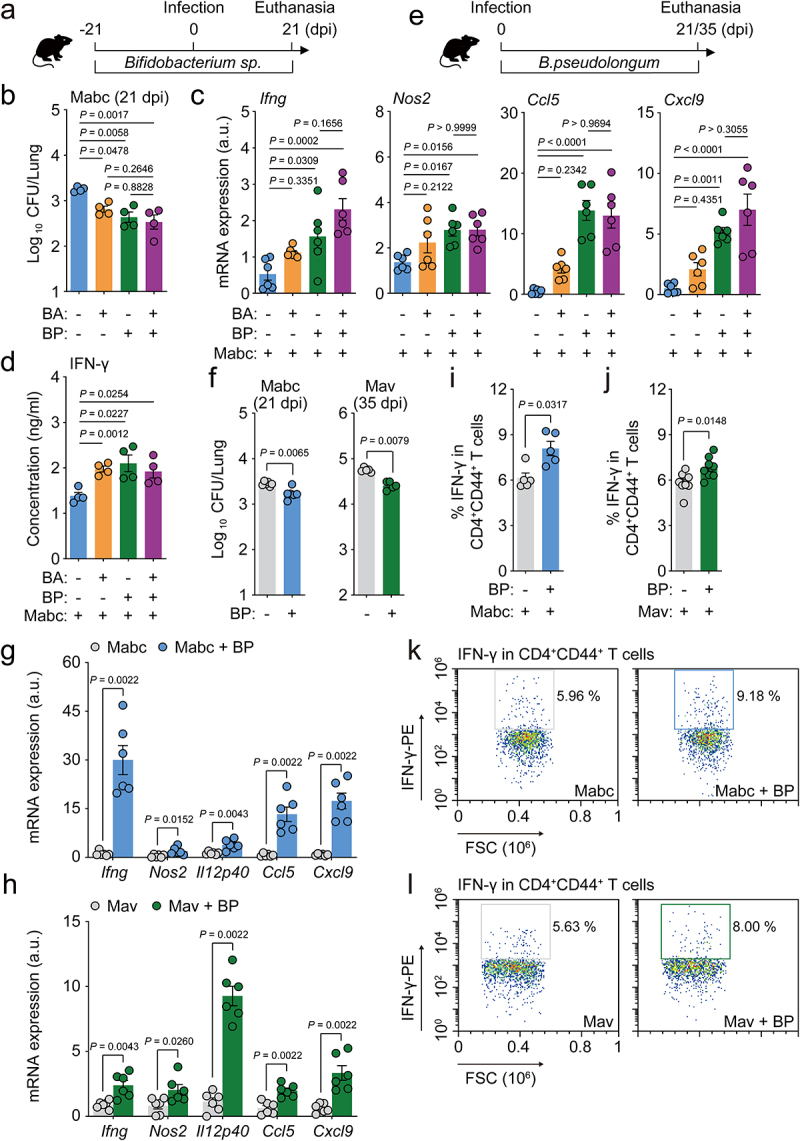
Oral administration of *Bifidobacterium* sp. enhances lung protective immunity during NTM-PD.

To investigate whether the selected *Bifidobacterium* species induced protective effects against NTM infection, we treated mice with either *B. animalis* or *B. pseudolongum* before and after Mabc infection for a total of 6 weeks, as shown in Figure 7a. We found that the *in vivo* pulmonary Mabc loads were significantly decreased following treatment with either species; there were no significant differences in the activation of antimicrobial responses between the two bacteria. In addition, combined treatment with *B. animalis* and *B. pseudolongum* did not cause any increase in the antimicrobial responses against Mabc infection, compared to those induced by either *Bifidobacterium* species alone (Figure 7b). Moreover, treatment with *B. pseudolongum*, but not *B. animalis*, significantly increased *Ifng, Nos2, Ccl5*, and *Cxcl9* mRNA levels in the lungs during Mabc infection (Figure 7c). The IFN-γ production level in the lungs was significantly higher in mice treated with *B. animalis, B. pseudolongum*, or both compared to the control mice (Figure 7d).

(**a**–**d**) Mice were subjected to pre- and post-administration of vehicle, *Bifidobacterium animalis*, *Bifidobacterium pseudolongum*, or a mixture of both bacteria by oral gavage for a total of 6 weeks. After 3 weeks of administration, mice were infected with Mabc and euthanized at 21 dpi. (**a**) Schematic diagram of the experimental design. (**b**) Lung bacterial loads (CFU) for four different groups of Mabc-infected mice (n = 4 mice per group). (**c**) *Ifng*, *Nos2*, *Ccl5*, and *Cxcl9* mRNA expression levels in lung tissues from mice determined by qRT-PCR (*n* = 6 mice per group). (**d**) IFN-γ concentrations in the supernatants of lung lysates from the different groups of mice measured with ELISA (*n* = 4 mice per group). (**e**–**l**) Mice were intranasally infected with Mabc or Mav and supplemented with vehicle or *B. pseudolongum* for 21 dpi (for Mabc) or 35 dpi (for Mav). (**e**) Schematic diagram of the experimental design. (**f**) Lung bacterial loads (CFU) from mice infected with Mabc (left) or Mav (right) at designated time points (*n* = 5 mice per group). (**g** and **h**) *Ifng*, *Nos2*, *Il12p40*, *Ccl5*, and *Cxcl9* mRNA expression levels in the lung tissues of mice infected with (**g**) Mabc or (**h**) Mav determined by qRT-PCR (*n* = 6 mice per group). (**i**–**l**) Flow cytometric analysis of cervical lymph nodes from Mabc- or Mav-infected mice treated with vehicle or *B. pseudolongum*. (**i** and **j**) The proportion of IFN-γ-positive CD4^+^CD44^+^ T cells from mice infected with (**i**) Mabc (*n* = 5 mice per group) or (**j**) Mav (*n* = 8 mice per group). (**k** and **l**) Representative dot plots of IFN-γ-positive CD4^+^CD44^+^ T cells from mice infected with (**k**) Mabc or (**l**) Mav. Statistical significance was calculated by one-way ANOVA with Tukey’s multiple comparison test (**b**, **c**), unpaired *t* test (**d**), and Mann–Whitney U test (**f**–**j**). Data are presented as the mean ± SEM and are representative of at least three independent experiments. a.u., arbitrary units; BA, *B. animalis*; BP, *B. pseudolongum*; CFU, colony forming units; dpi, days postinfection.

Given the observation that *B. pseudolongum* enhances antimicrobial and protective cytokine/chemokine expression in the lung tissues of infected mice, we examined the potential therapeutic effects of *B. pseudolongum* treatment on Mabc and Mav infection. We treated mice with *B. pseudolongum* for at least 3 weeks following Mabc or Mav infection (Figure 7e) and found significantly decreased *in vivo* bacterial loads in the lung tissues of these NTM-infected mice (Figure 7f). In addition, postinfection treatment with *B. pseudolongum* caused marked increases in the mRNA expression levels of *Ifng, Nos2, Il12p40, Ccl5*, and *Cxcl9* in the lungs of NTM-infected mice (Figure 7g,h). Treatment with *B. pseudolongum* significantly increased IFN-γ production in CD4^+^ effector T cell populations (Figure 7i–l) in cervical lymph nodes of NTM-infected mice compared to control mice.

**Figure 8.**
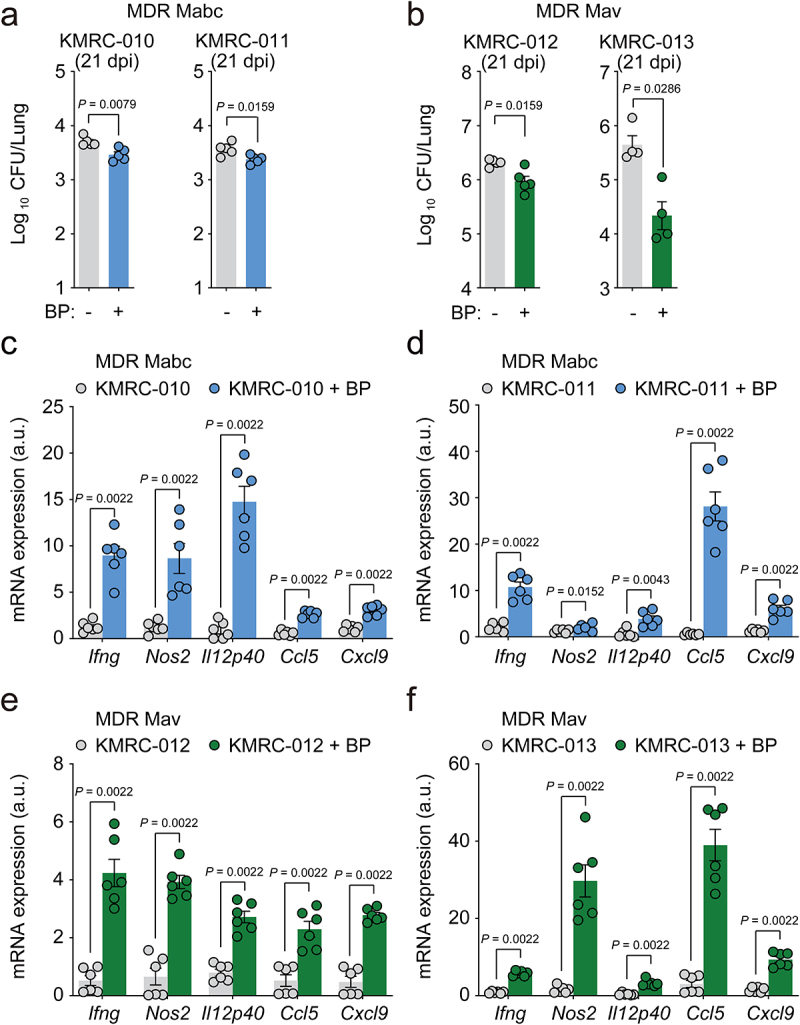
*Bifidobacterium pseudolongum* administration confers host pulmonary defense against multidrug-resistant (MDR) strains of NTM bacteria. Mice were intranasally infected with MDR clinical Mabc (KMRC-010 and −011) or Mav (KMRC-012 and −013) and treated with vehicle or *B. pseudolongum* for 21 days. Mice were euthanized at 21 dpi and the lungs were resected.

Furthermore, postinfection treatment with *B. pseudolongum* caused a significant decrease in the *in vivo* bacterial loads (Figure 8a,b), whereas it increased IFN-γ-related cytokine mRNA expression (Figure 8c–f) in the lung tissues of mice infected with four different clinical strains of either Mabc or Mav, both of which show multidrug resistance to clarithromycin, ethambutol, and rifampicin. Taken together, these data strongly suggest that oral administration of *B. pseudolongum* mimics the l-arginine-mediated protective host responses against NTM-PD and can effectively treat infections caused by multidrug-resistant clinical strains.

(**a** and **b**) Lung bacterial loads (CFU) from mice infected with (**a**) MDR-Mabc strains (KMRC-010 Mabc and KMRC-011 Mabc; *n* = 5 mice per group) or (**b**) MDR-Mav strains (KMRC-012 Mav and KMRC-013 Mav; *n* = 4-5 mice per group). (**c**–**f**) *Ifng, Nos2, Il12p40, Ccl5*, and *Cxcl9* mRNA expression levels in the lung tissues of mice infected with (**c**) KMRC-010 Mabc, (**d**) KMRC-011 Mabc, (**e**) KMRC-012 Mav, or (**f**) KMRC- 013 Mav determined using qRT-PCR (*n* = 6 mice per group). Statistical significance was determined by Mann–Whitney U test. Data are presented as the mean ± SEM and are representative of at least two independent experiments. a.u., arbitrary units; BP, *B. pseudolongum*; CFU, colony forming units; dpi, days postinfection.

### *The metabolite inosine, but not suberic acid, enhances antimicrobial responses against NTM infections* in vitro *and* in vivo

**Figure 9.**
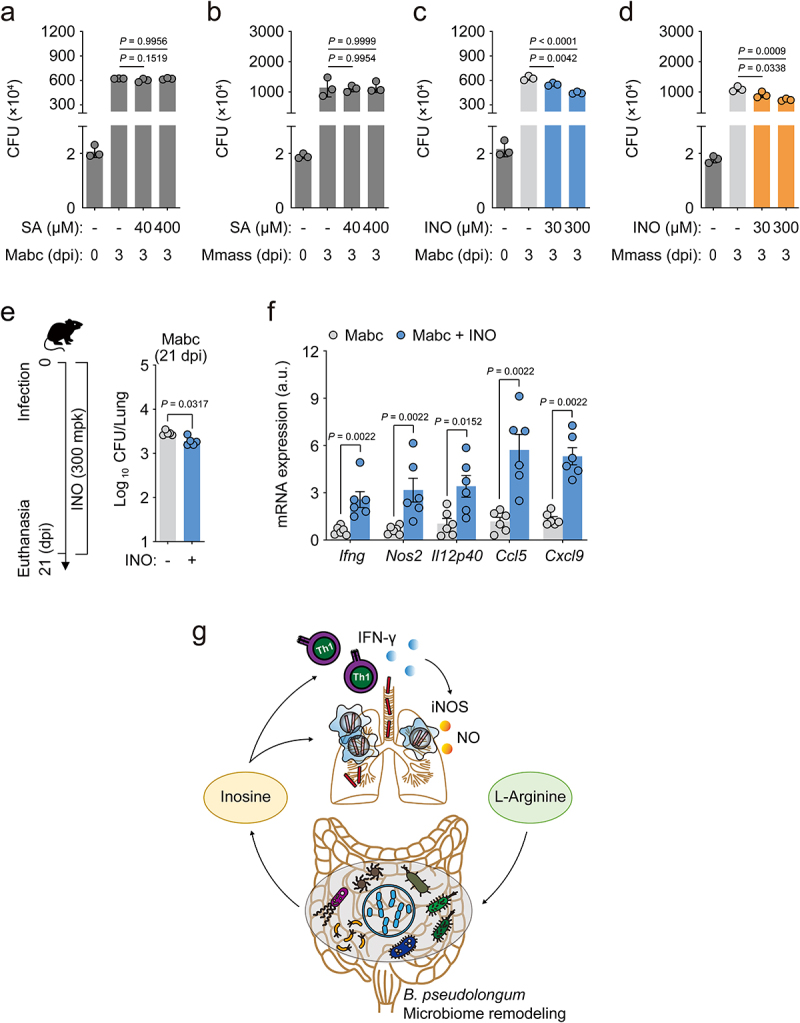
Inosine treatment enhances *in vitro* and *in vivo* antimicrobial responses against NTM infection.

Recent studies have shown that *B. pseudolongum* enhances the population of CD8^+^ T-bet^+^ T cells in the small intestine and induces Th1 differentiation and IFN-γ production, promoting antitumor immune responses by producing the purine metabolite inosine.^49^ In addition, *Bifidobacterium* treatment significantly increases the level of suberic acid in mouse serum.^50^ Given these previous observations, we considered the possibility that either inosine or suberic acid may be involved in host defense against NTM infection. Therefore, we infected bone marrow-derived macrophages (BMDMs) from C57BL/6 mice with Mabc or Mmass, and incubated them with or without treatment with either inosine or suberic acid for 3 days. Treatment of BMDMs with inosine, but not suberic acid, significantly inhibited intracellular mycobacterial survival in a dose-dependent manner (Figure 9a–d). Moreover, inosine treatment reduced *in vivo* bacterial loads but increased IFN-γ-related cytokine expression in the lung tissues of mice infected with Mabc (Figure 9e,f).

(**a**–**d**) Intracellular survival of NTM in bone marrow-derived macrophages (BMDMs) infected with Mabc or Mmass (MOI = 1) in the presence or absence of suberic acid (40 or 400 μM) or inosine (30 or 300 μM), measured via CFU analysis at 3 dpi. BMDMs were infected with **(a)** Mabc or (**b**) Mmass in the presence or absence of suberic acid, and (**c**) Mabc or (**d**) Mmass in the presence or absence of inosine (n = 3 mice per group). (**e** and **f**) Mice were intranasally infected with Mabc and administered vehicle or inosine (300 mg/kg) for 21 dpi. (**e**) Schematic diagram showing time points (left) and lung bacterial loads (CFU) of mice infected with Mabc at designated time points (right, n = 5 mice per group). (**f**) *Ifng*, *Nos2*, *Il12p40*, *Ccl5*, and *Cxcl9* mRNA expression levels in the lung tissues of mice infected with Mabc determined by qRT-PCR (n = 6 mice per group). (**g**) Schematic representation of the gut–lung axis microbiome model in NTM-PD proposed in this study. l-Arginine-induced remodeling of the gut microbiome contributes to pulmonary host defense against NTM infection, at least partly mediated through inosine, via Th1-mediated M1 shift. Statistical significance was calculated by one-way ANOVA with Tukey’s multiple comparison test (**a**–**d**) and Mann–Whitney U test (**e** right and **f**). Data are representative of at least three independent experiments and are presented as the mean ± SEM. a.u., arbitrary units; CFU, colony forming units; dpi, days postinfection; INO, inosine; SA, suberic acid.

We further examined whether inosine level was increased in the sera of l-arginine-treated mice (see Figure 2d). Targeted metabolomics analyses showed that the inosine level was significantly increased by l-arginine treatment in the sera from Mabc-infected mice, compared to those from either Mabc-infected or l-arginine-treated mice (Figure S6). These data strongly suggested that l-arginine administration led to increased inosine level *in vivo* and thus enhanced antimicrobial host defense against NTM infection. These observations suggested that oral administration of either *B. pseudolongum* or inosine promoted l-arginine-induced pulmonary host defense against NTM-PD through enhancement of protective M1 and Th1 immune responses (Figure 9g).

## Discussion

Effective host-directed therapies are urgently needed to treat NTM-PD, for which treatments are often complicated and ineffective.^1,8,9^ Previously, we found that the PBMCs of NTM patients infected with Mabc or Mmass had prominent inflammatory characteristics.^32^ In the present study, we showed that the l-arginine level was lower in the sera of NTM-PD patients and NTM-infected mice, and that oral administration of l-arginine significantly reduced *in vivo* NTM bacterial loads and enhanced protective IFN-γ-producing T cell responses and iNOS expression in mice. Furthermore, the gut microbiomes of l-arginine-treated mice were shifted toward *Bifidobacterium* sp.-dominant profiles, which conferred pulmonary immune defense against NTM-PD. Oral administration of *B. pseudolongum* significantly increased antimicrobial responses against NTM-PD, even against infection with multidrug-resistant clinical strains of Mabc or Mav. We further showed that the administration of inosine, a known metabolite of *B. pseudolongum*, showed similar antimicrobial activities against NTM-PD.

The two major l-arginine-converting enzymes, i.e., iNOS and Arg1, are involved in the production of NO and the urea cycle, respectively.^34,51^ M1 macrophage-driven iNOS metabolizes l-arginine into NO and citrulline, thus promoting antimicrobial activity.^34^ Comparison of the metabolomes in serum collected from NTM-PD patients and HCs revealed markedly lower concentrations of l-arginine, but not of citrulline, in patients infected with Mabc or Mmass. By contrast, the serum urea level was significantly higher in NTM-PD patients than HCs, suggesting an M2 shift in NTM patients. Similarly, mice infected with Mabc showed an increased serum urea level after 3 weeks of infection. Importantly, pre- and postinfection oral treatment of NTM-infected mice with l-arginine significantly decreased *in vivo* bacterial loads and pathological inflammation in the lung tissues. Previous studies have shown that NTM patients exhibit attenuated Th1/Th17 differentiation capacity^52^ and a dysregulated IFN-γ/IL-12B axis, the critical immune pathway associated with NTM infection.^9^ Therefore, our data strongly suggest that l-arginine treatment during NTM infection confers beneficial effects by upregulation of M1 macrophage responses, probably driven by IFN-γ-producing protective T lymphocytes.

The results of the present study indicate that l-arginine-mediated gut microbiota remodeling promotes host pulmonary immune defense against NTM lung infection. The gut–lung axis contributes to the modulation of host defense against various diseases. The crosstalk between the gut and lungs, in part via their microbiomes, bidirectionally influences immune responses.^53^ A recent study showed that oral administration of *Lactobacillus plantarum* CIRM653 to mice intranasally infected with *Klebsiella pneumoniae* significantly ameliorated the lung inflammation with reduced infiltration of macrophages and neutrophils.^17^ *L. plantarum* administration significantly suppresses the production of proinflammatory cytokines and chemokines in bronchoalveolar fluid and increases regulatory T cell response in the lungs of *K. pneumoniae*-infected mice.^17^ Taken together with our findings, these data strongly suggest that gut microbiota can control remote immune modulation in the context of respiratory bacterial infection. In addition, a recent meta-analysis indicated that probiotics and prebiotics could effectively ameliorate clinical symptoms and mortality rates in patients with respiratory viral infections.^54^ Importantly, aging, a significant risk factor for NTM infection,^55,56^ is closely associated with changes in the gut–lung axis, and thus contributes to age-related susceptibility to respiratory diseases.^57^ Taken together, these observations highlight the critical involvement of the gut–lung axis in the pathogenesis of NTM lung infections and the potential utility of beneficial microbes for the future development of novel therapeutics against pathogenic respiratory infection.

Our findings from the FMT and ABX treatment experiments suggest that the gut microbiota is critically involved in l-arginine-mediated protective immune defenses against NTM lung infections. Moreover, oral administration of either *B. pseudolongum* or *B. animalis* increased pulmonary antimicrobial responses and the expression of IFN-γ-related cytokines and Th1 responses during infections caused by rapidly growing Mabc and slowly growing Mav. Although the function of *B. pseudolongum* in the context of pathogenic bacterial infection is unclear, our data were partly consistent with the recent finding that oral administration of *B. animalis* alone or in combination with *B. pseudolongum* significantly suppressed the disease severity of H7N9 influenza pulmonary infection.^58^ Several studies have emphasized the function of *B. pseudolongum* in activating Th1 immune functions and ameliorating pathological inflammation in various disease models in a context-dependent manner. For example, *B. pseudolongum* plays a critical role in enhancing the efficacy of immune checkpoint inhibitors in different cancer models.^49^ Mechanistically, intestinal *B. pseudolongum* increases the expression of T-bet, the Th1 master transcriptional factor, in CD4^+^ T cells of the small intestinal lamina propria, but not in the spleen.^49^ In addition, oral gavage of *B. pseudolongum* significantly reduces the inflammatory and fibrotic responses in cardiac allografts.^59^ In this model, the immunomodulatory effects of *B. pseudolongum* are partly mediated by induction of the antiinflammatory cytokine, IL-10, and inhibition of proinflammatory cytokines, TNF-α and IL-6.^59^ Taken together with our findings that l-arginine ameliorated pathological lung inflammation, these data strongly suggest that oral *Bifidobacterium* sp. play a major role in promoting Th1 responses and controlling excessive lung inflammation.

Due to significant limitations in using current antibiotics and the increasing incidence of antibiotic resistance in pathogenic infections over the last two decades, there are urgent medical needs to develop novel host-directed therapeutics against infectious diseases.^60–62^ NTM infections are often fatal in immunocompromised patients and treatment is particularly challenging because of multiple antibiotic resistance.^61,62^ Importantly, we found that *B. pseudolongum* administration reduced *in vivo* bacterial loads during pulmonary infections with multidrug-resistant clinical strains of Mabc. Given the controversial findings of clinical trials investigating the efficacy of arginine supplementation upon TB outcomes,^28–30^ our findings warrant further evaluation of gut microbiota composition in patients before treatment with arginine. Taken together, these data strongly suggest the potential of *B. pseudolongum* as a preventive and therapeutic intervention for chronic NTM-PD patients infected with drug-resistant strains.

As suggested by recent findings of the metabolites produced by *B. pseudolongum*,^49,50^ we found that the purine metabolite, inosine, but not suberic acid, mimicked *B. pseudolongum*-induced antimicrobial host defense against NTM infection. Our data were partly consistent with previous findings that *B. pseudolongum*-induced inosine upregulates Th1 differentiation and IFN-γ production, which are potentially related to antitumor immune responses.^49^ It remains to be determined how inosine treatment significantly increased antimicrobial responses in macrophages. As an early study showed that inosine inhibits the production of proinflammatory cytokines in macrophages and spleen cells,^63^ the antimicrobial effects of inosine may have been due to the activation of distinct antimicrobial pathways rather than the regulation of inflammatory cytokines. Further studies are required to obtain additional insights into the detailed mechanisms by which inosine treatment upregulates antimicrobial responses in macrophages infected with NTMs.

In conclusion, this study demonstrated that the gut–lung axis is critically involved in l-arginine-induced host protection against NTM-PDs. l-Arginine administration triggered pulmonary host defense against NTM infection by remodeling the intestinal microbiota toward enrichment with *B. pseudolongum*, activating protective immune responses. Taken together, these data will facilitate the development of new therapeutic approaches targeting the gut microbiota and their metabolites to enhance antimicrobial pulmonary immunity during NTM-PD, even against infection with multidrug-resistant clinical strains.

## Materials and methods

### Ethics approval and consent to participate

Patients’ clinical and laboratory data were obtained from an ongoing prospective observational cohort study to investigate NTM pulmonary disease, approved by Institutional Review Board (clinicaltrials.gov identifier NCT00970801). The Research and Ethics Committee of Chungnam National University Hospital, Daejeon, Korea, approved this study (202009A-CNU-155). Mice were treated in accordance with the guidelines of the Institutional Animal Care and Use Committee, Chungnam National University School of Medicine, Daejeon, Korea (CNUH-A0011-1), and the Korean Food and Drug Administration.

### Human samples

Plasma and PBMC samples from NTM-infected patients or HCs were obtained from an existing cohort of patients, as described previously.^32^ NTM in human patients was identified as reported in our previous study.^32^ The baseline characteristics of the 16 Mabc-infected and 16 Mmass-infected patients are presented in Table 1. There were no significant differences in characteristics between the two patient groups. The nCounter assay was performed in 11 Mabc-infected patients, 7 Mmass-infected patients, and 6 HCs. All subjects were female and of similar age (*P* = 0.31, Kruskal–Wallis test). The mean ages of the Mabc-infected patients, Mmass-infected patients, and HCs were 61.1, 59.4, and 55.2 years, respectively.^32^

### Metabolite extraction, LC-MS analysis, and processing of human serum samples

Sera metabolome samples were extracted as reported previously.^64^ Briefly, aliquots of 50 µL (approx. 2–2.5 mg total protein) of sera were treated with 1 µL proteinase K (AM2546; Invitrogen) and 1 µL 250 mM CaCl_2_ (C1016; Sigma-Aldrich) for 30 min at 37°C to release associated metabolites by relaxing the native protein structure. After incubation, the samples were treated with 150 µL ice-cold acetonitrile (3:1, v:v), completely resuspended, and stored at – 20°C for longer than 1 h. The samples were clarified by centrifugation and then filtered across a 0.22 µm spin-X column (8169; Costar).

LC-MS differentiation and detection of extracted metabolites were performed using an Agilent Accurate Mass 6230 TOF coupled with an Agilent 1290 Liquid Chromatography system. The metabolites were separated on a Congent Diamond Hydride Type C column (gradient 3) (Microsolve Technologies) with solvents and configuration as reported previously.^65^ The mobile phase consisted of solution A (ddH_2_O with 0.2% formic acid) and solution B (acetonitrile with 0.2% formic acid). An isocratic pump was used for continuous infusion of a reference mass solution to allow mass axis calibration. Detected ions were deemed to be metabolites on the basis of unique accurate mass-retention time identifiers for masses exhibiting the expected distribution of accompanying isotopologs. The abundance of extracted metabolites was analyzed using Profinder B06.00 software and Agilent Qualitative Analysis B.07.00 with a mass tolerance of <0.005 Da. The clustered heatmap, hierarchical clustering, principal component analysis, and fold change analysis were performed using bioinformatics tools available in MetaboAnalyst v.5.0. (www.metaboanalyst.ca). Univariate statistical analysis by one-way ANOVA for multiple comparisons was used to identify significant differences in the abundances of metabolites between groups.

### Nanostring nCounter assay and data analysis

Human PBMCs were isolated from heparinized venous blood using Lymphoprep (1114545; Axis-shield) in accordance with the manufacturer’s instructions. Total RNA from PBMCs was extracted using TRIzol reagent (15596026; Invitrogen) in accordance with the manufacturer’s instructions, followed by RNA quantification and assessment using QIAxpert (Qiagen). Nanostring nCounter Human Immunology gene expression assays for PBMCs were performed at PhileKorea Technology (Daejeon, South Korea), and the data were analyzed by nSolver version 4.0 (NanoString Technologies) as described previously.^32^

### Preparation of NTM strains

Mabc (ATCC 19977) and Mav (ATCC 25291) were obtained from American Type Culture Collection (ATCC). Mmass (KMRC-00136-13018) and the multidrug-resistant strains of Mabc (KMRC-00800-00010 and −00011) and Mav (KMRC-00800-00012 and −00013) were obtained from the Korean Mycobacterium Resource Center (KMRC). The multidrug-resistant strains of Mabc and Mav showed resistance to clarithromycin, ethambutol, and rifampicin. Bacteria were cultured and prepared as single-cell suspension as follows. NTM strains were incubated at 37°C with shaking in Middlebrook 7H9 medium (271310; BD Bioscience) containing 10% oleic albumin dextrose catalase (OADC) until mid-log phase (OD_600_ = 0.4). Bacteria in mid-log phase (OD_600_ = 0.4) were used in all experiments as described previously.^66^ After bacterial culture, the pellets were harvested by centrifugation at 500 × *g* for 20 min and washed with phosphate-buffered saline (PBS) three times to remove bovine serum albumin (BSA). After adding glass beads 1 mm in diameter and PBS, the precipitated pellet was homogenized using a Mixer mill 200 bead beater (Buch & Holm) for 4 min at 20 Hz. The homogenized suspensions were filtered using a 40 μm cell strainer to remove undispersed bacterial clumps. Finally, 1 mL aliquots of the bacterial single-cell suspensions were stored frozen at –80°C until use.

### Mycobacterial infection and assessment of bacterial load

Frozen bacterial cells were thawed, centrifuged, and the pellet was resuspended in PBS containing 0.05% Tween 80 (PBST). Mice were anesthetized and infected by intranasal administration of bacteria (Mabc, Mmass, Mav, and KMRC-011 at 1 × 10^7^ CFU/mouse and KMRC-010, −012, and −013 at 1 × 10^6^ CFU/mouse). At the indicated times after NTM infection, mice were euthanized, and the lungs were removed to assess the bacterial load. Lung tissues were homogenized in PBST using a tissue homogenizer (OMNI TH). Serial dilutions of the homogenates were plated in 7H10 agar plates, and colonies were counted after incubation at 37°C for 3–4 or 10–12 days in the case of rapidly growing and of slowly growing mycobacteria, respectively.

### Maintenance and treatment of mice

C57BL/6 mice (3–8 weeks old) were purchased from Samtako Bio, and were maintained under a 12 h light/dark cycle under specific-pathogen-free conditions. Age- and sex-matched animals were randomly assigned to drug or vehicle treatment groups. l-Arginine (A5131; Sigma-Aldrich) was dissolved in drinking water (vehicle control) at a concentration of 3.75 mg/mL and provided before and/or after the infection, as indicated in the figure legends. Inosine (I4125; Sigma-Aldrich) was dissolved in PBS at a concentration of 30 mg/mL and given to mice daily at a dose of 300 mg/kg by gavage after infection, as indicated in the figure legends.

For *Bifidobacterium* supplementation, the type strains of *Bifidobacterium pseudolongum* (KCTC 3224) and *Bifidobacterium animalis* (KCTC 3219) were cultured under anaerobic conditions in Lactobacilli MRS agar (288210; BD Difco) supplemented with 0.5% (w/v) l-cysteine hydrochloride at 37°C. Mice were given either *B. pseudolongum* or *B. animalis* orally at 10^8^ CFU suspended in 200 µL PBS (vehicle) once a day for 6 weeks, from 3 weeks before infection to 3 or 5 weeks after infection, as shown in Figure 7a,e.

For ablation of bacteria from the intestine, mice were orally treated with an antibiotic mixture consisting of 1 g/L of ampicillin sodium USP (AMP25B; Ambrothia), 1 g/L neomycin trisulfate salt hydrate (N6386; Sigma-Aldrich), 1 g/L gentamicin solution (G1397; Sigma-Aldrich), 1 g/L metronidazole (M3761; Sigma-Aldrich), 0.5 g/L vancomycin hydrochloride (V2002; Sigma-Aldrich), and 0.1 g/L amphotericin B (1397–89-3; Duchefa-Biochemie) dissolved in sterilized water for 3 days from 5 days before infection.

### Fecal sample collection and fecal microbiota transplantation

For the fecal microbiota transplantation (FMT) experiments, fresh feces was collected from mice fed l-arginine (3.75 mg/mL in drinking water) or vehicle (drinking water) for 3 weeks. Aliquots of pooled fecal slurry from each group were immediately stored at –80°C until use for FMT. Before FMT, about 20 mg feces (2–3 fecal pellets) was resuspended in 2 mL PBS and the solution was vigorously mixed with a homogenizer. After centrifugation at 1500 rpm for 3 min, the supernatant (about 1 mL) was collected and 200 µL was administered per mouse immediately by oral gavage three times per week. Treatment was continued for 7 weeks, from 21 days before to 28 days after infection.

### Mouse serum metabolome analysis

Metabolite extraction and metabolome analysis were performed by MetaMass (Seoul, Korea). Briefly, 100 µL serum was added to 1 mL methanol containing internal standard (2-chloro-phenylalanine, 1 mg/mL). The mixture was vortexed for 1 min and sonicated for 10 min. Each sample was broken up using an MM400 mixer mill (Retsch) at a frequency of 30 Hz for 10 min, and the extracts were incubated at 4°C for 1 h. Then the extracts were centrifuged at 13,000 rpm for 10 min at 4°C, and the supernatants were collected. Supernatants were filtered using 0.2 μm polytetrafluoroethylene (PTFE) syringe filters (Chromdisc) and dried using a speed-vacuum concentrator (Biotron). The dried samples were processed in two steps of derivatization reaction before GC-TOF/MS analysis. Oximation was conducted first by adding 50 µL methoxyamine hydrochloride in pyridine (20 mg/mL) to the dried samples, and the reaction mixture was incubated at 30°C for 90 min. Subsequently, silylation was performed using adding 50 µL *N*-trimethylsilyl-*N*-methyl trifluoroacetamide (MSTFA) to the incubated reaction mixture, followed by incubation at 37°C for 30 min. All samples were filtered through a PTFE syringe filter before analysis. GC-TOF/MS analysis was performed using an Agilent 7890A system (Agilent Technologies) with an L-PAL3 autosampler and Pegasus III TOF-MS (Leco Corp.). The GC-TOF/MS raw data were obtained by MetaMass (Seoul, Korea) using LECO Chroma TOF™ software (version 4.44; Leco Corp.), and converted into the NetCDF format (*.cdf). Peak detection, peak intensity normalization, retention time evaluation, and alignment were performed using the Metalign software package (http://www.metalign.nl) and exported to an Excel file (Microsoft Corp.). Multivariate statistical analyses were performed using SIMCA-P+ (version 12.0; Umetrics). Partial least squares-discriminant analysis (PLS-DA) modeling was performed to compare the different metabolites between experimental groups. The significantly discriminant variables among experimental groups were selected based on a variable importance in projection (VIP) value >1.0 and tested for significance at *p* < 0.05. Inosine in mouse serum was detected by targeted metabolomics using GC-TOF/MS by MetaMass (Seoul, Korea) and confirmed based on the retention time and mass (m/z).

### Lung tissue RNA-Seq analysis

Total RNA was isolated from the lung tissue using TRIzol reagent (15596018; Invitrogen) and a PureLink RNA Mini Kit (12183018A; Invitrogen) in accordance with the manufacturer’s instructions. Total RNA concentration was calculated by Quant-IT RiboGreen (R11490; Invitrogen). The integrity of total RNA was assessed by running the samples on the TapeStation RNA ScreenTape (5067–5576; Agilent). Only high-quality RNA preparations with RIN more significant than 7.0 were used for RNA library construction. A library was independently prepared with 1 µg total RNA for each sample using an Illumina TruSeq Stranded mRNA Sample Prep Kit (RS-122-2101; Illumina Inc.). The first step in the workflow involved purifying the poly(A)-containing mRNA molecules using poly(T)-attached magnetic beads. Following purification, the mRNA was fragmented into small pieces using divalent cations under elevated temperature. The cleaved RNA fragments were copied into first-strand cDNA using SuperScript II reverse transcriptase (18064014; Invitrogen) and random primers. This was followed by second-strand cDNA synthesis using DNA Polymerase I, RNase H, and dUTP. Then, these cDNA fragments were end-repaired, a single A base was added, followed by adapter ligation. Then the products were purified and enriched by PCR to generate the final cDNA library. The libraries were quantified using KAPA Library Quantification Kits for Illumina Sequencing platforms according to the qPCR Quantification Protocol Guide (KK4854; Kapa Biosystems) and qualified using TapeStation D1000 ScreenTape (5067–5582; Agilent Technologies). Then the indexed libraries were submitted to an Illumina NovaSeq (Illumina Inc.), and paired-end (2 × 100 bp) sequencing was performed by Macrogen Inc. (Seoul, South Korea). We used the Snakemake^67^ workflow to process the raw reads; FastQC (version 0.11.9) was used to preprocess and control the quality of raw reads;^68^ Cutadapt-PE (version 2.1) was used to trim the adapter sequence from paired-end reads;^69^ Trimmed reads in FASTQ format were quantified at the transcript level using Salmon^70^ against a reference mouse genome (GRCm38 March 2020). The results of transcript quantification were imported to the gene level with the tximport package (version 1.20), and differential expression of genes was analyzed using the DESeq2 package^71^ (version 1.32) in R (version 4.0.5). Genes were considered significantly differentially expressed at adjusted *P* < 0.05 (Benjamini–Hochberg [BH] multiple test correction method). The z-scores were calculated for each gene row using the average gene counts of biological replicates and were visualized as a heatmap using Excel 2019 (Microsoft Corp.).

### RNA extraction and qRT-PCR

Total RNA from mouse lung tissue homogenates was extracted using TRIzol reagent (15596026; Invitrogen) in accordance with the manufacturer’s instructions, followed by RNA quantification and assessment using QIAxpert (Qiagen). cDNA was synthesized from total RNA using reverse transcription master premix (EBT-1543; ELPIS Biotech) in accordance with the manufacturer’s instructions. Real-time PCR was performed using SYBR Green reagent (204074; Qiagen) in a real-time PCR Cycler Rotor-Gene Q 2plex system (9001620; Qiagen). The mRNA expression was calculated with the 2^ΔΔ^ threshold cycle (Ct) method, with normalization relative to *Gapdh*. The sequences of primers used in this study are shown in Table S2.

### Histological analysis

After resecting the lungs from mice, tissues were fixed in 10% formalin and embedded in paraffin. Paraffin blocks were cut into sections 4 µm thick and stained with hematoxylin and eosin for light microscopic examination. Quantification of the inflamed area was assessed by scanning the whole lung tissue fields, and the MFI of the red threshold from split channels was determined using FIJI software.^72^

### Immunofluorescence

Sections of mouse lung tissue 3 µm thick were prepared for immunofluorescence staining using primary antibodies against inducible nitric oxide synthase (iNOS; NOS2) (sc-7271; Santa Cruz Biotechnology), F4/80 (sc-52664; Santa Cruz Biotechnology), and appropriate secondary antibodies (A11029 and A21209; Invitrogen). Nuclei were stained with 4′,6-diamidino-2-phenylindole (DAPI) (P36935; Invitrogen). Immunofluorescence images were acquired using a Leica TCS SP8 microscope system (Leica).

### Flow cytometry

After 3 weeks of NTM infection, mice were euthanized by asphyxiation with CO_2_, and cervical lymph nodes were resected to prepare single-cell suspensions (1 × 10^6^ cells). Cells were stimulated with 50 ng/mL phorbol-12-myristate-13-acetate (PMA) (P8139; Sigma) and 1 µM ionomycin (I0634; Sigma) for 2 h followed by addition of Golgistop (554715; BD Biosciences) for 4 h at 37°C. Then the cells were harvested and washed three times with PBS. Staining for cell-surface markers was also performed using APC-Cy7-conjugated anti-CD3 (557596, 1:250; BD Biosciences), Brilliant Violet (BV) 605-conjugated anti-CD4 (563151, 1:250; BD Biosciences), and V450-conjugated anti-CD44 (560451, 1:250; BD Biosciences) antibodies at 4°C for 30 min. After washing three times with PBS, cells were fixed and permeabilized using a Cytofix/Cytoperm Kit (554715; BD Biosciences) 4°C for 30 min. Cells were washed three times with Perm/Wash (BD Biosciences) and stained intracellularly with PE-conjugated anti-IFN-γ antibody (554412, 1:250; BD Biosciences) at 4°C for 30 min. Stained cells were resuspended in PBS and immediately assayed on a Novocyte 2060R (Acea Biosciences). Data were collected and analyzed using FlowJo software (BD Biosciences).

### Enzyme-linked immunosorbent assay

Mouse lung lysates were collected and stored at –80°C. Samples were diluted and analyzed using a Mouse IFN-γ ELISA kit (555138; BD Biosciences) in accordance with the manufacturer’s instructions.

### Isolation of microbial DNA and 16S rRNA gene-based amplicon sequencing

Mouse fecal samples were collected, and DNA for metagenomic analysis was extracted using a QIAamp DNA Stool Mini Kit (51504; Qiagen) as described previously.^73^ Hypervariable regions (V3–V4) of the 16S rRNA gene were amplified by PCR using bacterial universal primers: 341F (5′-TCGTCG-GCA-GCG-TCA-GAT-GTG-TAT-AAG-AGA-CAGCCT-ACG-GGN-GGC-WGC-AG-3′) and 805R (5′-GTC-TCG-TGG-GCT-CGG-AGA-TGT-GTA-TAAGAG-ACA-GGA-CTA-CHV-GGG-TAT-CTA-ATC-C-3′). PCR was performed using C1000 and S1000 thermal cyclers (Bio-Rad) with an initial denaturation step at 95°C for 3 min followed by 24 cycles of 95°C for 30 s, 55°C for 30 s, and 72°C for 30 s, with a final step at 72°C for 5 min. Triplicate PCR reactions with the same DNA template were pooled and purified using a QIAquick PCR Purification kit (28106; Qiagen). Purified amplicon sizes were calculated, and amplicons were sequenced on an Illumina MiSeq platform using a 2 × 300 bp reagent kit for paired-end sequencing by Macrogen Inc. (Seoul, South Korea).

### Processing and analysis of sequencing data

The 16S rRNA gene amplicon sequencing data were imported into and analyzed on the QIIME2 (version 2020.8) platform,^74^ and paired reads were quality filtered and demultiplexed. After importing and demultiplexing, sequences were denoised using the DADA2 algorithm^75^ in the QIIME2 package and trimmed based on the sequencing quality plot (median quality score >30) using the parameters (--p-trim-left-f [15] --p-trim-left-r [10] --p-trunc-len-f [284] --p-trunc-len-r [213]). Amplicon sequence variants (ASVs) present in <3 samples were removed, and data were rarefied to the lowest sample read count using the parameter (--p-sampling-depth 52000). High-quality ASVs were assigned for taxonomic classification with the QIIME feature-classifier classify-sklearn trained against the 99% clustered 16S rRNA reference sequence set of the Greengenes database version 13.8.^76^ Within-sample diversity was estimated using Chao1 index,^77^ an estimator of richness; Shannon diversity index,^78^ which integrates ASV richness and abundance into a single evenness value; Fisher’s index,^79^ which calculates the relationship between the abundance of each species and the number of species; and Faith’s phylogenetic diversity whole tree,^80^ which is based on a phylogenetic tree and the sum of all of the branch lengths as a measure of diversity. Beta diversity was calculated based on unweighted UniFrac distance and weighted UniFrac distance by PCoA. LEfSe analysis was conducted to confirm the bacterial taxa at the genus level that could be used to discriminate between the experimental groups.^81^ For prediction of bacterial functional pathways, the PICRUSt2 pipeline^82^ version 2.3.0b was used, with the table of ASV counts and ASV sequences as the input using the full pipeline script command (picrust2_pipeline.py). Pathway abundances of each sample, predicted by MetaCyc, were visualized by PCoA plot, based on binary-Jaccard distance^83^ using QIIME2 (version 2020.8),^74^ and significantly abundant pathways were identified by LEfSe with LDA score >2.0.

### BMDM preparation, infection, and intracellular CFU assays

Bone marrow from wild-type mice was harvested and cultured in Dulbecco’s modified Eagle’s medium (DMEM) (12–604 F; Lonza) containing 10% fetal bovine serum (FBS) (16000–044; Gibco) for 3–5 days in the presence of 25 ng/mL macrophage colony-stimulating factor (416-ML; R&D Systems). Fully differentiated BMDMs were infected with Mabc or Mmass (MOI = 1) for 2 h. Extracellular bacteria were removed by washing with PBS. BMDMs were further cultured in the presence or absence of suberic acid (40 and 400 μM) or inosine (30 and 300 μM) for 3 days. To analyze intracellular bacterial viability, infected cells were lysed in distilled water to release intracellular bacteria. The serially diluted homogenates of the infected cells were plated on 7H10 agar plates, and colonies were counted after 3–4 days of incubation.

### Statistical analysis

All data were analyzed using GraphPad Prism (version 8.0.2 or 8.4.3) and are presented as the mean ± standard error of the mean (SEM). In all analyses, *P* < 0.05 was taken to indicate statistical significance. Outliers for comparison of relative abundance were eliminated by the ROUT (robust regression and outlier removal) method using Prism with the Q coefficient value set to 1%. Statistical comparisons were performed using the Mann–Whitney U-test, unpaired *t* test, or one-way ANOVA with Tukey’s multiple comparison test, as appropriate. Significant differences in PLS-DA were determined by analysis of variance (ANOVA) using PASW Statistics 18 software (SPSS). Box plots were rendered using the relative peak area of unique metabolite masses by STATISTICA 7 software (StatSoft Inc.), and the data were analyzed by multiple *t* tests with Holm–Sidak correction for multiple comparisons using GraphPad Prism. All reported results were replicable. The number of animals and the number of replicates for each experiment are described in the figure legends.

## Supplementary Material

Supplemental Material

## Data Availability

All of the sequencing data that support the findings of the study have been deposited in the NCBI under accession code GSE176281 and PRJNA735090. Command for the 16S rRNA gene amplicon sequencing and RNA-seq analysis were provided through supplementary materials.
