# Supplementary material for: Arginine-mediated gut microbiome remodeling promotes host pulmonary immune defense against nontuberculous mycobacterial infection
Source: Gut Microbes. 2022 May 17;14(1):2073132. doi: 10.1080/19490976.2022.2073132 (PMC9116420; doi:10.1080/19490976.2022.2073132)
Supplement: Supplemental Material [file KGMI_A_2073132_SM0118.docx]

**Supplemental file for:**

**Arginine-mediated gut microbiome remodeling promotes host pulmonary immune defense against nontuberculous mycobacterial infection**

**Running Title:** Arginine-induced microbiota remodeling in host defense

**Supplementary Figures and Figure Legends**


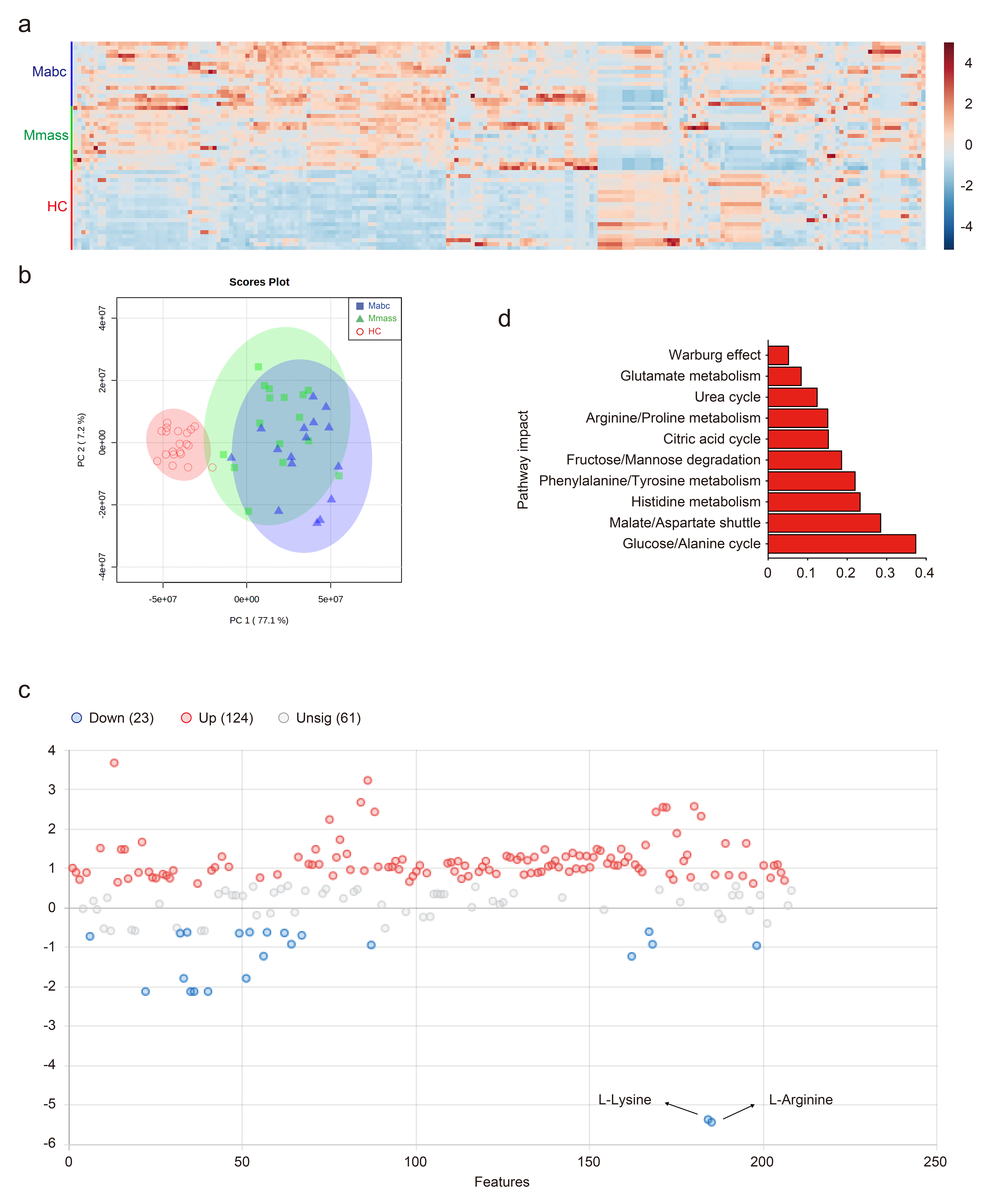


**Figure S1.** Altered metabolic pathways in NTM-PD patients' sera. Untargeted metabolomics analysis and accompanied pathway enrichment assays of sera from HCs (*n* = 20) or Mabc- (*n* = 16) and Mmass- (*n* = 16) infected patients. **(a)** Clustered heatmap depicting levels of ~210 metabolites. Columns indicate individual metabolites. Data were parsed using uncentered Pearson's correlation with centroid linkage clustering and rendered using the image generation program MetaboAnalyst (ver. 5.0). Data are depicted on a log2 scale. **(b)** A 2-dimensional principal component analysis (PCA) plot reveals separation in metabolite profiles altered in NTM-PD patients' sera compared to that of HCs. **(c)** Scatter plot of log2 transformed fold change showing the ratio of the mean abundance in NTM-PD patients' sera to that of HCs' sera. Metabolites that are significantly upregulated and downregulated are depicted by red dots and blue dots, respectively. Metabolites that are not significantly changed are denoted by gray dots. **(d)** Statistically over-represented pathways in NTM-PD patients' sera relative to HCs' sera were screened by pathway enrichment analysis. Top-ranked pathways are described, and Arginine/Proline pathway and the Urea cycle are identified.


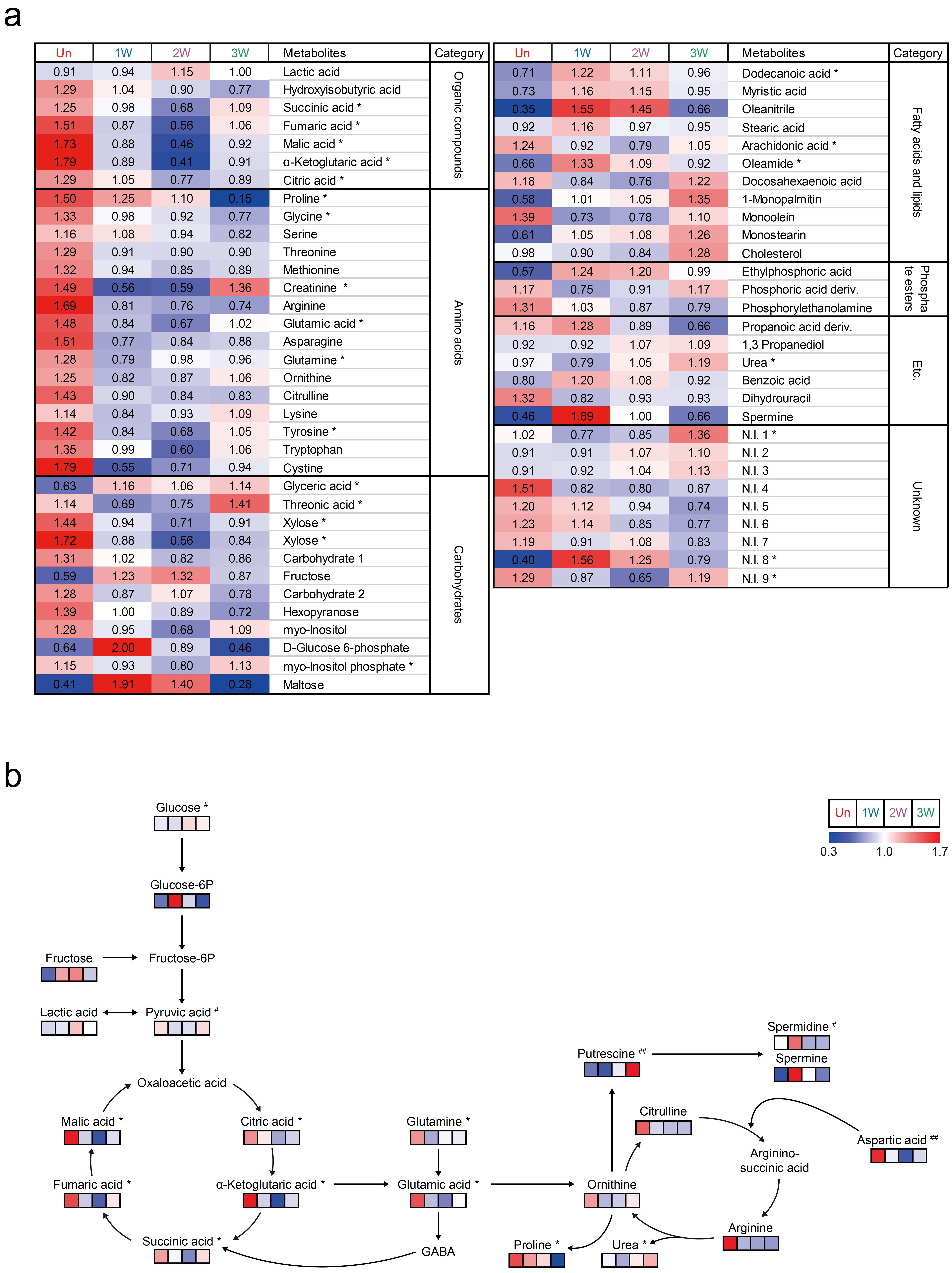


**Figure S2.** Metabolomics analysis of serum contents from the mice during Mabc infection. The results of serum metabolomics analysis from the uninfected and Mabc-infected (1w, 2w, and 3w post-infection groups) Mabc. **(a)** Heatmap analysis of the relative abundance of significantly different serum metabolites (VIP score > 1.0) based on the PLS-DA model. **(b)** The metabolic pathway of identified metabolites in mouse serum. The pathway was adjusted from the KEGG database. Heatmap and colored squares (blue-to-red) in the pathway represent fold changes normalized by each metabolite level in the experimental group. Statistical significance was determined by One-way ANOVA between the experimental group. **p* < 0.05.


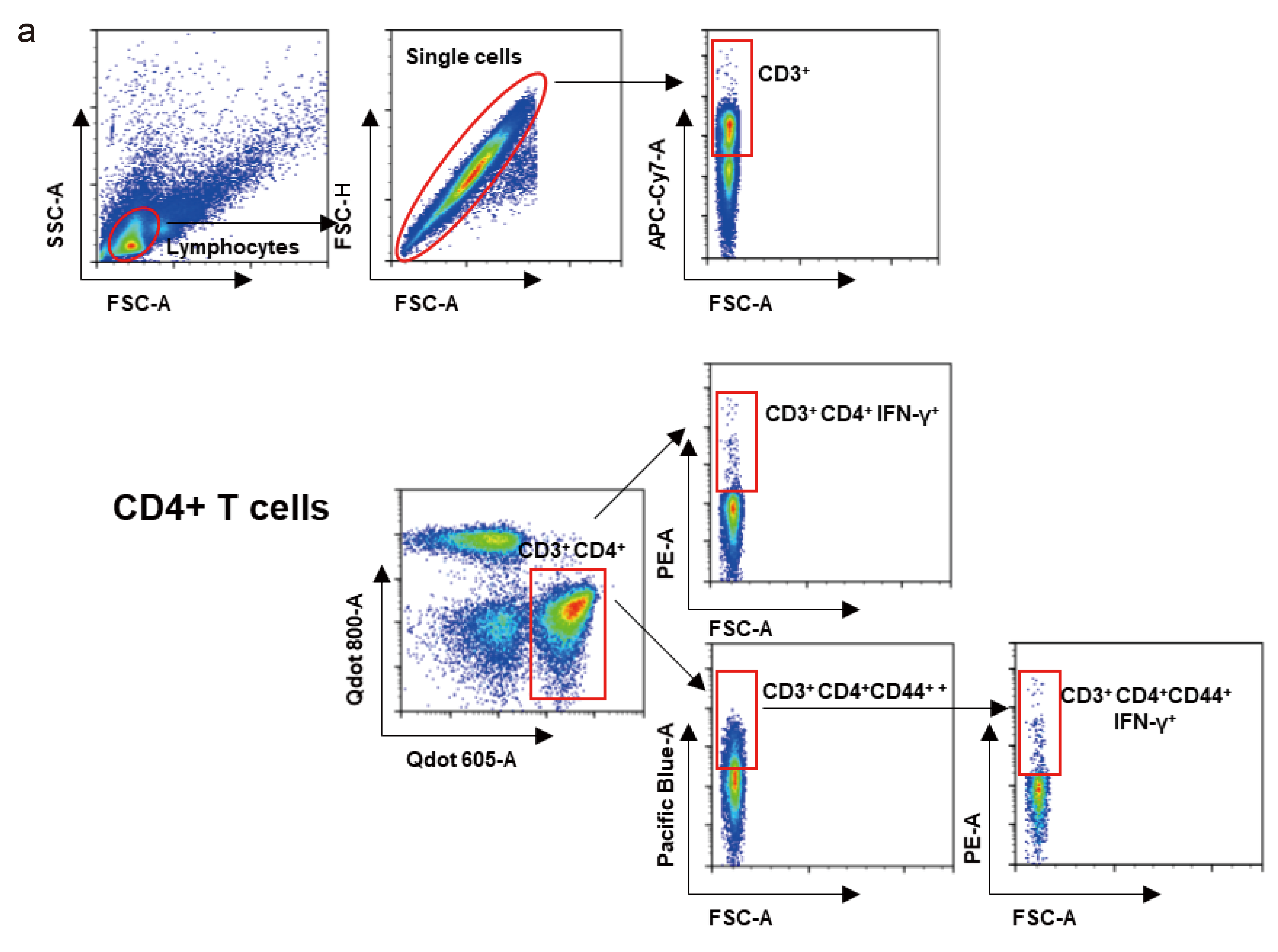


**Figure S3**. An illustrating example of the flow cytometric gating strategy used to identify distinct populations for nucleated cells in this study. Lymphocytes were first determined by a low forward scatter (FSC), and low side scatter (SSC) gate with murine cervical lymph nodes from the NTM-PD mice model. The lymphocytes were gated again in an FSC-A and FSC-H dot plot for the elimination of doublets, and were stained with CD3 (APC-Cy7) as the T cell markers. CD4+ T lymphocytes were gated in a CD4 (Qdot 605) and CD8 (Qdot 800) dot plot, as shown in the bottom-left panel. CD44 (Pacific Blue) was stained for the activated and memory T cells marker, and IFN-γ (PE-A) was stained for the effective cytokines to eliminate NTM.


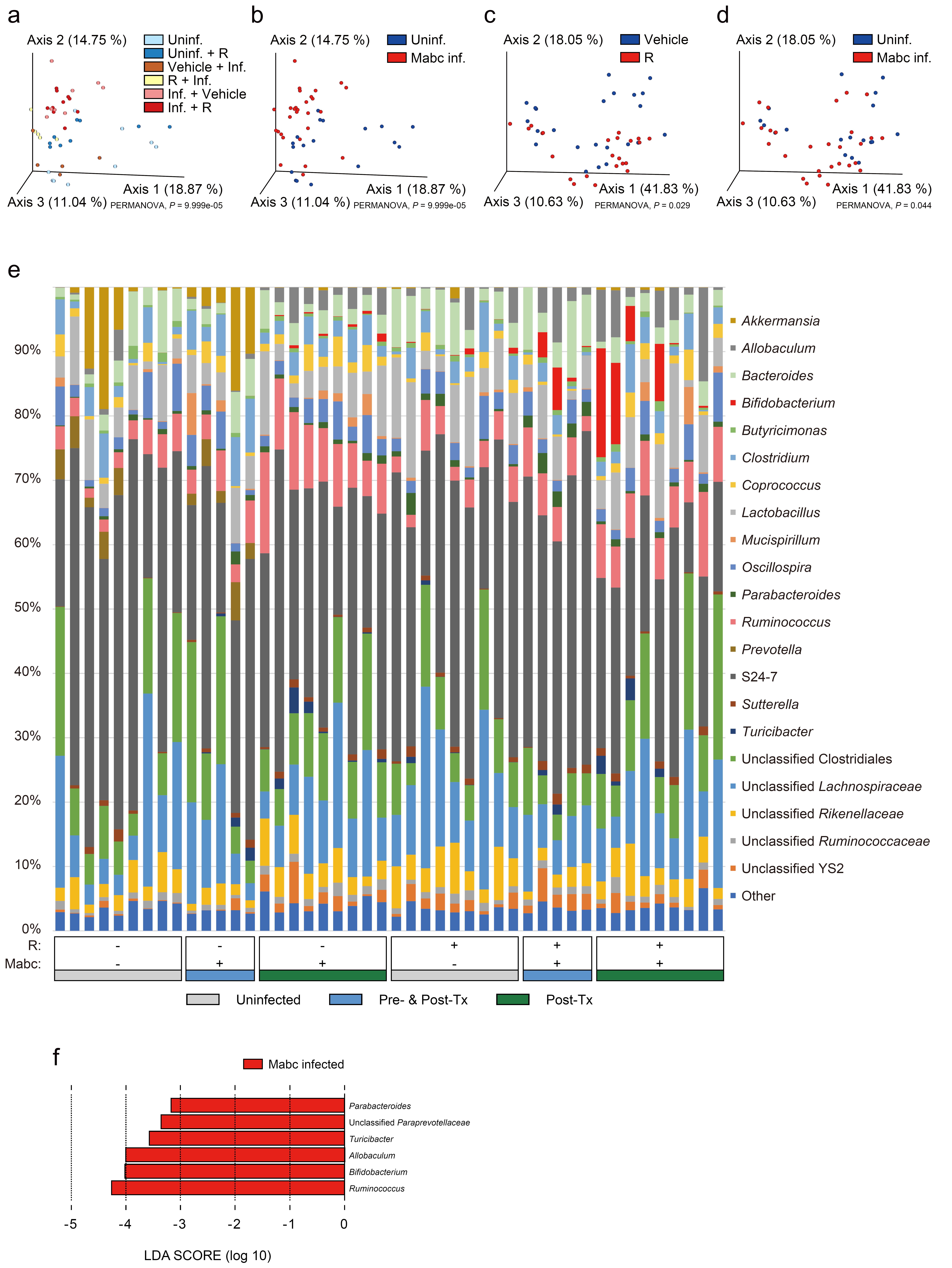


**Figure S4.** l-Arginine treatment and Mabc infection altered gut microbiota composition. The feces were collected from six experimental groups and were analyzed by 16S rRNA amplicon sequencing (n = 9 mice in uninfected and Post-tx group; n = 5 mice in Pre- & Post-tx group). **(a)** Principal coordinate analysis (PCoA) plot based on unweighted UniFrac distance metrics representing the significant differences in six experimental groups (Permanova, *P* = 9.999e-05). **(b)** PCoA plot based on unweighted UniFrac distance metrics representing the significant differences in fecal microbiota structure by Mabc infection in mice (*P* = 9.999e-05). **(c and d)** PCoA plot based on weighted UniFrac distance metrics representing the significant differences in fecal microbiota structure by (c) l-arginine (R) treatment (*P* = 0.029) or (d) Mabc infection (*P* = 0.044). **(e)** Genus-level taxonomic composition of gut microbiota for each experimental group. **(f)** Significantly enriched bacterial taxa in Mabc infected mice were identified by linear discriminant analysis (LDA) effect size (LEfSe) analysis (LDA score > 3.0, *P* < 0.05). The PERMANOVA with 999 permutations determined statistical significance (a to d).


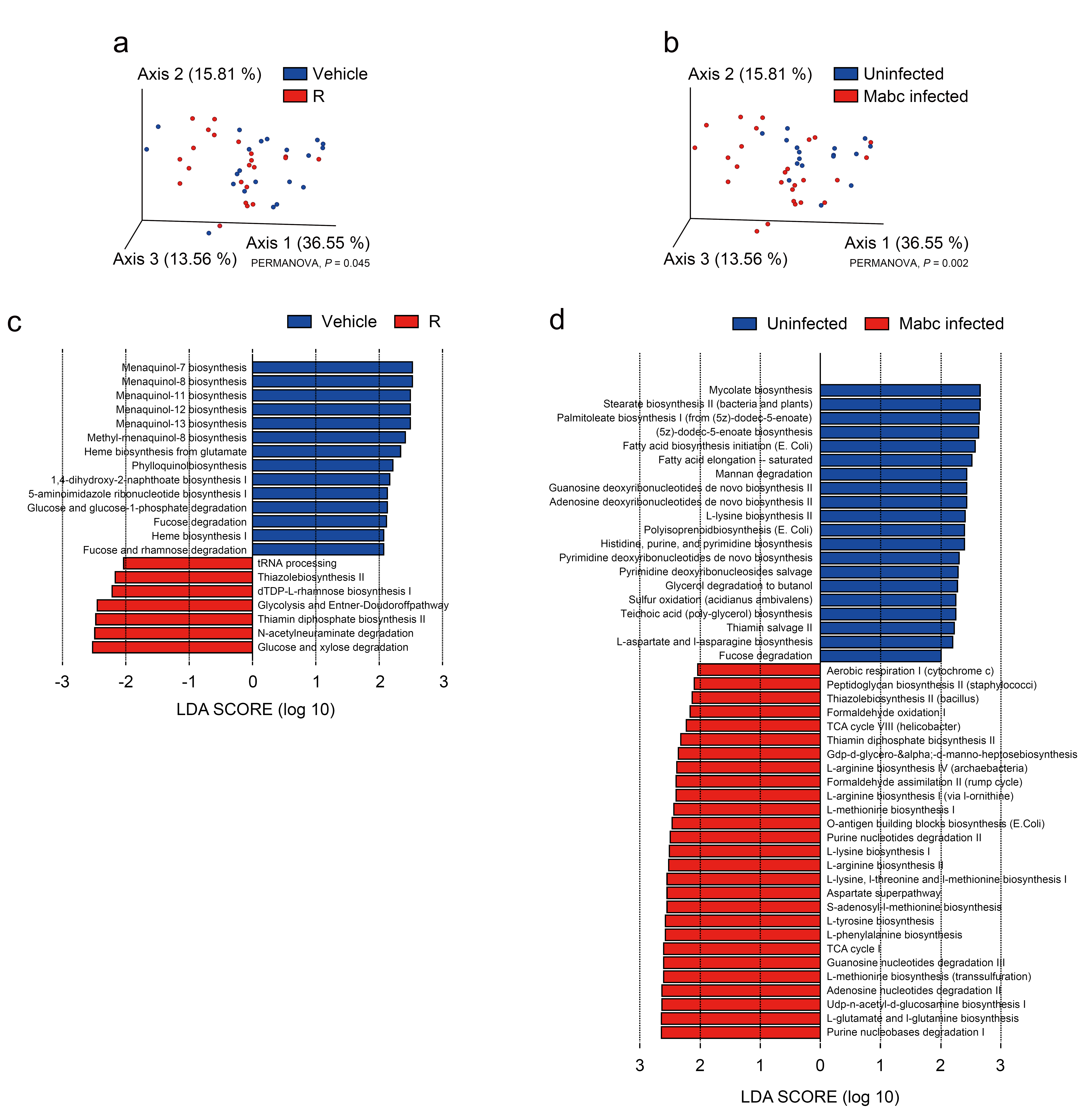


**Figure S5.** l-Arginine treatment and Mabc infection altered gut microbial functional pathways**.** The abundances of MetaCyc pathways were predicted with samples from Figure S4. (**a** and **b**) PCoA plot based on binary Jaccard distances showed the differences in predicted functional pathway by (a) l-arginine (R) treatment (*P* = 0.045) or (b) Mabc infection (*P* = 0.002). **(c)** Differentially enriched functional pathways between vehicle and l-arginine (R) -treated mice were identified by LEfSe analysis (LDA score > 2.0, *P* < 0.05). **(d)** Differentially enriched functional pathways between Mabc-uninfected and Mabc-infected mice were identified by LEfSe analysis (LDA score > 2.0, *P* < 0.05). Statistical significance was determined by the PERMANOVA with 999 permutations (a and b).

**Figure S6.** l-Arginine treatment in Mabc-infected mice increased the serum inosine concentration. The results of targeted metabolomics analysis of sera from the uninfected or Mabc-infected mice in the presence or absence of l-arginine supplementation were represented by a barplot and compared value of peak area (intensity). Statistical significances were calculated using an unpaired *t*-test. Error bars denote ± SEM. *, *P* < 0.05; ***, *P* < 0.001.

**Supplementary Tables**

| **Table S1. Significantly changed metabolites between HC and Mabc/Mass** | | | |  |
| --- | --- | --- | --- | --- |
|  | Metabolite name | Fold Change | log2(FC) | |
| **Up-regulated** | 11H-Benzbcaceanthrylene | 1.5311 | 0.61452 | |
|  | 4-Amino-2-hydroxylamino-6-nitrotoluene | 1.5316 | 0.61507 | |
|  | Triethylene glycol diglycidyl ether | 1.5666 | 0.64767 | |
|  | PE(20:0/15:0) | 1.5821 | 0.6618 | |
|  | Pantothenic Acid | 1.6156 | 0.69208 | |
|  | L-Proline | 1.642 | 0.71544 | |
|  | 1-5'-Phosphoribosyl-5-formamido-4-imidazolecarboxamide | 1.6426 | 0.71596 | |
|  | PC(20:3,8Z,11Z,14Z/14:0) | 1.6638 | 0.73449 | |
|  | 5-Methylangelicin | 1.6675 | 0.73768 | |
|  | N-Phenyl-1-naphthylamine | 1.6795 | 0.748 | |
|  | NAC-Diketide | 1.6812 | 0.74949 | |
|  | Chlorphenesin | 1.6906 | 0.75751 | |
|  | Icaceine | 1.6964 | 0.7625 | |
|  | Allamandin | 1.701 | 0.76635 | |
|  | Ketoleucine | 1.708 | 0.77227 | |
|  | PC(20:21,1Z,14Z/14:0) | 1.7417 | 0.80052 | |
|  | PC(18:0/14:0) | 1.7418 | 0.80054 | |
|  | 3,5-Dinitrosalicylic acid | 1.7517 | 0.80872 | |
|  | Propionic acid | 1.7581 | 0.81399 | |
|  | Formononetin | 1.762 | 0.81723 | |
|  | L-Histidine | 1.7702 | 0.82391 | |
|  | D-Mannitol | 1.7856 | 0.83643 | |
|  | PC(22:5,7Z,10Z,13Z,16Z,19Z/14:0) | 1.7893 | 0.8394 | |
|  | Aconine | 1.7932 | 0.84256 | |
|  | Mukaadial | 1.8055 | 0.85238 | |
|  | Levulinic acid | 1.8114 | 0.85711 | |
|  | PE(22:4,7Z,10Z,13Z,16Z/P-16:0) | 1.815 | 0.85994 | |
|  | PC(22:4,7Z,10Z,13Z,16Z/14:0) | 1.8382 | 0.87827 | |
|  | PE(22:2,13Z,16Z/14:0) | 1.8385 | 0.8785 | |
|  | L-Tryptophan | 1.8547 | 0.8912 | |
|  | PC(22:2,13Z,16Z/14,19Z) | 1.8547 | 0.8912 | |
|  | 2D-Mannosyl-D-glycerate | 1.8555 | 0.8918 | |
|  | Bupropion | 1.859 | 0.89456 | |
|  | Inosine | 1.8615 | 0.89647 | |
|  | L-Methionine | 1.8732 | 0.90549 | |
|  | Saphenic acid | 1.8785 | 0.9096 | |
|  | PE22113Z/150 | 1.8815 | 0.91187 | |
|  | PC2264Z,7Z,10Z,13Z,16Z,19Z/1619Z | 1.8845 | 0.9142 | |
|  | PC22213Z,16Z/140 | 1.8856 | 0.91504 | |
|  | PE2048Z,11Z,14Z,17Z/P-160 | 1.8934 | 0.92096 | |
|  | Lactosylceramide d181/120 | 1.8964 | 0.92328 | |
|  | Succinic acid | 1.9195 | 0.94073 | |
|  | Doxepin | 1.9268 | 0.94618 | |
|  | Tiocarbazil | 1.9282 | 0.94724 | |
|  | PE2257Z,10Z,13Z,16Z,19Z/P-160 | 1.9423 | 0.95776 | |
|  | PG160/160 | 1.95 | 0.96346 | |
|  | PG160/1819Z | 1.9625 | 0.97266 | |
|  | PC2035Z,8Z,11Z/1829Z,12Z | 1.9732 | 0.98055 | |
|  | PC2264Z,7Z,10Z,13Z,16Z,19Z/160 | 1.9904 | 0.99308 | |
|  | L-Valine | 1.9956 | 0.99683 | |
|  | Urea | 2.0101 | 1.0072 | |
|  | Ethopropazine | 2.0338 | 1.0242 | |
|  | PC2247Z,10Z,13Z,16Z/160 | 2.0346 | 1.0247 | |
|  | PE2264Z,7Z,10Z,13Z,16Z,19Z/P-180 | 2.0358 | 1.0256 | |
|  | PC1839Z,12Z,15Z/140 | 2.0404 | 1.0289 | |
|  | Jatrophone | 2.0534 | 1.038 | |
|  | GABA | 2.055 | 1.0392 | |
|  | PC20111Z/140 | 2.0576 | 1.041 | |
|  | PE20211Z,14Z/150 | 2.058 | 1.0412 | |
|  | PE24115Z/150 | 2.0697 | 1.0494 | |
|  | PG180/1829Z,12Z | 2.0941 | 1.0663 | |
|  | L-Phenylalanine | 2.0986 | 1.0694 | |
|  | guanabenz | 2.1044 | 1.0734 | |
|  | PC2045Z,8Z,11Z,14Z/2038Z,11Z,14Z | 2.105 | 1.0738 | |
|  | PC1846Z,9Z,12Z,15Z/150 | 2.1091 | 1.0766 | |
|  | PC2264Z,7Z,10Z,13Z,16Z,19Z/1819Z | 2.1117 | 1.0784 | |
|  | Oligomycin C | 2.1239 | 1.0867 | |
|  | Stigmatellin Y | 2.1309 | 1.0915 | |
|  | 5-hydroxy valeric acid | 2.1343 | 1.0938 | |
|  | L-Tyrosine | 2.1364 | 1.0952 | |
|  | Mycinamicin VII | 2.1473 | 1.1025 | |
|  | makisterone B | 2.1565 | 1.1087 | |
|  | PC2264Z,7Z,10Z,13Z,16Z,19Z/1839Z,12Z,15Z | 2.1743 | 1.1205 | |
|  | PC2048Z,11Z,14Z,17Z/140 | 2.1898 | 1.1308 | |
|  | PC1419Z/20211Z,14Z | 2.2217 | 1.1516 | |
|  | PC2264Z,7Z,10Z,13Z,16Z,19Z/180 | 2.2284 | 1.156 | |
|  | PC1839Z,12Z,15Z/P-160 | 2.2637 | 1.1787 | |
|  | PE220/150 | 2.2696 | 1.1824 | |
|  | PC1829Z,12Z/140 | 2.272 | 1.1839 | |
|  | 3-Demethylubiquinone-9 | 2.274 | 1.1852 | |
|  | PE2264Z,7Z,10Z,13Z,16Z,19Z/P-1819Z | 2.2853 | 1.1924 | |
|  | PC2048Z,11Z,14Z,17Z/P-160 | 2.2999 | 1.2016 | |
|  | PC2264Z,7Z,10Z,13Z,16Z,19Z/140 | 2.3271 | 1.2185 | |
|  | PE20111Z/150 | 2.3385 | 1.2256 | |
|  | PC2264Z,7Z,10Z,13Z,16Z,19Z/1829Z,12Z | 2.3999 | 1.263 | |
|  | R-Lactate | 2.4223 | 1.2764 | |
|  | PE24115Z/1419Z | 2.4233 | 1.277 | |
|  | PC22213Z,16Z/1619Z | 2.4309 | 1.2815 | |
|  | PC1819Z/00U | 2.4381 | 1.2857 | |
|  | PC2038Z,11Z,14Z/P-160 | 2.4403 | 1.287 | |
|  | PC2257Z,10Z,13Z,16Z,19Z/180 | 2.4467 | 1.2908 | |
|  | PE2247Z,10Z,13Z,16Z/180 | 2.4517 | 1.2938 | |
|  | 2-Hydroxyethylphosphonate | 2.4553 | 1.2959 | |
|  | PC2055Z,8Z,11Z,14Z,17Z/P-160 | 2.4616 | 1.2996 | |
|  | PC2247Z,10Z,13Z,16Z/P-160 | 2.4808 | 1.3108 | |
|  | PC2038Z,11Z,14Z/150 | 2.4816 | 1.3113 | |
|  | PC2257Z,10Z,13Z,16Z,19Z/P-160 | 2.4951 | 1.3191 | |
|  | Coenzyme Q9 | 2.5336 | 1.3412 | |
|  | Calafatimine | 2.5748 | 1.3644 | |
|  | PC2264Z,7Z,10Z,13Z,16Z,19Z/P-160 | 2.6158 | 1.3873 | |
|  | PE24115Z/P-1819Z | 2.7247 | 1.4461 | |
|  | PS(18:0/20:0) | 2.7815 | 1.4759 | |
|  | Dibenzofuran | 2.7862 | 1.4783 | |
|  | Capillin | 2.7862 | 1.4783 | |
|  | Hydrocortisone cypionate | 2.7927 | 1.4816 | |
|  | PE(24:11,5Z/18:4,6Z,9Z,12Z,15Z | 2.7992 | 1.485 | |
|  | PC(22:6,4Z,7Z,10Z,13Z,16Z,19Z/P-18:0) | 2.807 | 1.489 | |
|  | 1-Hydroxy-2-naphthoic acid | 2.8502 | 1.511 | |
|  | D-Ribose 5-phosphate | 3.0067 | 1.5882 | |
|  | N-acetylaspartate | 3.0974 | 1.6311 | |
|  | 7-Cyano-7-deazaguanine | 3.1004 | 1.6324 | |
|  | Fluoren-9-one | 3.1737 | 1.6661 | |
|  | Glyceric acid | 3.3095 | 1.7266 | |
|  | L-Glutamate | 3.6981 | 1.8868 | |
|  | Pyruvic acid | 4.7125 | 2.2365 | |
|  | Fructose | 4.9932 | 2.32 | |
|  | Hypoxanthine | 5.3781 | 2.4271 | |
|  | Threonate | 5.3838 | 2.4286 | |
|  | 4-Oxoproline | 5.8192 | 2.5408 | |
|  | Oxoglutaric acid | 5.8437 | 2.5469 | |
|  | Diallyl disulfide | 5.9329 | 2.5687 | |
|  | Succinic acid semialdehyde | 6.3701 | 2.6713 | |
|  | R-Malate | 9.3575 | 3.2261 | |
|  | S-Succinyldihydrolipoamide | 12.735 | 3.6707 | |
| **Down-regulated** | L-Arginine | 0.023236 | -5.4275 | |
|  | L-Lysine | 0.024328 | -5.3612 | |
|  | Acalyphin | 0.23004 | -2.12 | |
|  | Metofluthrin | 0.23004 | -2.12 | |
|  | Elephantopin | 0.23006 | -2.1199 | |
|  | Nitrendipine | 0.23009 | -2.1197 | |
|  | Pyriminobac-methyl | 0.28979 | -1.7869 | |
|  | Bisacodyl | 0.28979 | -1.7869 | |
|  | Glutamine | 0.4265 | -1.2294 | |
|  | Salvinorin A | 0.42784 | -1.2249 | |
|  | 5-Acetylamino-6-formylamino-3-methyluracil | 0.51534 | -0.95641 | |
|  | D-Erythrose | 0.52078 | -0.94126 | |
|  | Purine | 0.52726 | -0.9234 | |
|  | 13-Deoxycarminomycin | 0.52808 | -0.92116 | |
|  | N-Methyltryptamine | 0.60622 | -0.72209 | |
|  | Aspulvinone H | 0.61692 | -0.69684 | |
|  | Chitobiose | 0.63969 | -0.64455 | |
|  | Grandidentatin | 0.63974 | -0.64444 | |
|  | Oplophorus luciferin | 0.64325 | -0.63655 | |
|  | Neolinustatin | 0.65044 | -0.62052 | |
|  | N-Methyl-2,3,7,8-tetramethoxy-5,6-dihydrobenzophenathridine-6-ethanoic acid | 0.65046 | -0.62047 | |
|  | Losartan | 0.65184 | -0.61741 | |
|  | 2-C-Methyl-D-erythritol 4-phosphate | 0.65749 | -0.60495 | |

**Table S2.** Sequences of primers used in this study.

| Species | Target | Forward (5’-3') | Reverse (5’-3') |
| --- | --- | --- | --- |
| *Mus musculus* | *Arg1* | CTCCAAGCCAAAGTCCTTAGAG | AGGAGCTGTCATTAGGGACATC |
|  | *Ccl2* | TGACCCCAAGAAGGAATGGG | ACCTTAGGGCAGATGCAGTT |
|  | *Ccl5* | CCTGCTGCTTTGCCTACCTCTC | ACACACTTGGCGGTTCCTTCGA |
|  | *Cxcl2* | CATCCAGAGCTTGAGTGTGACG | GGCTTCAGGGTCAAGGCAAACT |
|  | *Cxcl9* | AACGTTGTCCACCTCCCTTC | CACAGGCTTTGGCTAGTCGT |
|  | *Gapdh* | CATCACTGCCACCCAGAAGACTG | ATGCCAGTGAGCTTCCCGTTCAG |
|  | *Ifng* | CGGCACAGTCATTGAAAGCC | TGCATCCTTTTTCGCCTTGC |
|  | *Il12p40* | TTGAACTGGCGTTGGAAGCACG | CCACCTGTGAGTTCTTCAAAGGC |
|  | *Il1b* | TACGGACCCCAAAAGATGA | TGCTGCTGCGAGATTTGAAG |
|  | *Il6* | TAGTCCTTCCTACCCCAATTTCC | TTGGTCCTTAGCCACTCCTTC |
|  | *Nos2* | GGTGAAGGGACTGAGCTGTTA | TGAAGAGAAACTTCCAGGGGC |
|  | *Tnf* | ACGGCATGGATCTCAAAGAC | AGATAGCAAATCGGCTGACG |

**Analysis command scripts**

**QIIME2 analysis using 16S rRNA gene sequencing data**

#activating qiime2 environment

conda activate qiime2-2020.8

#greengene 341F-805R classifier using q2 classifier

1) qiime tools import --type 'FeatureData[Sequence]' --input-path taxonomy_classifier/gg_13_8_otus/rep_set/99_otus.fasta --output-path taxonomy_classifier/gg_13_8_99_repset.qza

2) qiime tools import --input-path taxonomy_classifier/gg_13_8_otus/taxonomy/99_otu_taxonomy.txt --output-path taxonomy_classifier/gg_13_8_99_taxa.qza --type 'FeatureData[Taxonomy]' --input-format HeaderlessTSVTaxonomyFormat

3) qiime feature-classifier extract-reads --i-sequences taxonomy_classifier/gg_13_8_99_repset.qza --p-f-primer CCTACGGGNGGCWGCAG --p-r-primer GACTACHVGGGTATCTAATCC --p-trunc-len 450 --p-min-length 100 --p-max-length 600 --o-reads taxonomy_classifier/gg_13_8_99_341_805.qza

4) qiime feature-classifier fit-classifier-naive-bayes --i-reference-reads taxonomy_classifier/gg_13_8_99_341_805.qza --i-reference-taxonomy taxonomy_classifier/gg_13_8_99_taxa.qza --o-classifier taxonomy_classifier/gg_13_8_341_805_classifer.qza

#importing data

qiime tools import --type 'SampleData[PairedEndSequencesWithQuality]' --input-path [manifest.txt] --output-path [1.paired-end-demux.qza] --input-format PairedEndFastqManifestPhred33V2

#Demultiplexing sequences summary (qza->qzv)

qiime demux summarize --i-data 1.paired-end-demux.qza --o-visualization 1.paired-end-demux_summary.qzv

# Sequence quality control and feature table construction

qiime dada2 denoise-paired --i-demultiplexed-seqs 1.paired-end-demux.qza --p-trim-left-f 15 --p-trim-left-r 10 --p-trunc-len-f 284 --p-trunc-len-r 213 --o-table 2.table_15f284_10r213.qza --o-representative-sequences 2.rep-seqs_15f284_10r213.qza --o-denoising-stats 2.denoising-stats_15f284_10r213.qza --p-n-threads 64

#File format (qza to qzv)

qiime metadata tabulate --m-input-file 2.denoising-stats_15f284_10r213.qza --o-visualization 2.denoising-stats_15f284_10r213.qzv

#FeatureTable and FeatureData summaries

qiime feature-table summarize --i-table 2.table_15f284_10r213.qza --o-visualization 2.table_15f284_10r213.qzv --m-sample-metadata-file metadata.txt

qiime feature-table tabulate-seqs --i-data 2.rep-seqs_15f284_10r213.qza --o-visualization 2.rep-seqs_15f284_10r213.qzv

#ASV feature filtering

qiime feature-table filter-features --i-table 2.table_15f284_10r213.qza --p-min-frequency 20 --p-min-samples 3 --o-filtered-table 3.table_15f284_10r213_minfreq20_minsample3.qza

qiime feature-table summarize --i-table 3.table_15f284_10r213_minfreq20_minsample3.qza --o-visualization 3.table_15f284_10r213_minfreq20_minsample3.qzv --m-sample-metadata-file metadata.txt

# Generate a tree for phylogenetic diversity analyses

qiime phylogeny align-to-tree-mafft-fasttree --i-sequences 2.rep-seqs_15f284_10r213.qza --o-alignment 5.aligned_15f284_10r213.qza --o-masked-alignment 5.masked-aligned_15f284_10r213.qza --o-tree 5.unrooted-tree_15f284_10r213.qza --o-rooted-tree 5.rooted-tree_15f284_10r213.qza --p-n-threads 64

[pipeline: Build a phylogenetic tree using fasttree and mafft alignment]

#Rarefy table (using 3.SHP-table_10f263_10r218_minfreq20_minsample3.qza)

qiime feature-table rarefy --i-table 3.table_15f284_10r213_minfreq20_minsample3.qza --p-sampling-depth 52000 --o-rarefied-table 4.table_15f284_10r213_minfreq20_minsample3_subsampled52000.qza

# Generate visual and tabular summaries of a feature table (rarefy check)

qiime feature-table summarize --i-table 4.table_15f284_10r213_minfreq20_minsample3_subsampled52000.qza --o-visualization 4.table_15f284_10r213_minfreq20_minsample3_subsampled52000.qzv --m-sample-metadata-file metadata.txt

#Applies a collection of diversity metrics to a feature table.

qiime diversity core-metrics-phylogenetic --i-phylogeny 5.rooted-tree_15f284_10r213.qza --i-table 4.table_15f284_10r213_minfreq20_minsample3_subsampled52000.qza --p-sampling-depth 52000 --m-metadata-file metadata.txt --output-dir core-metrics-results/ --p-n-jobs-or-threads [64]

#compares categories

qiime diversity beta-group-significance --i-distance-matrix core-metrics-results/unweighted_unifrac_distance_matrix.qza --m-metadata-file metadata.txt --m-metadata-column treatment --p-method permanova --p-permutations 999 --o-visualization Argtreatment_unweighted.qzv

#alpha-diversity

qiime diversity alpha --i-table 4.table_15f284_10r213_minfreq20_minsample3_subsampled52000.qza --p-metric Chao1 --o-alpha-diversity core-metrics-results/chao1.qza

#Alpha diversity qza to qzv

qiime diversity alpha-group-significance --m-metadata-file metadata.txt --i-alpha-diversity core-metrics-results/faith_pd_vector.qza --o-visualization faith_pd.qzv

# taxonomic classification

qiime feature-classifier classify-sklearn --i-reads 2.rep-seqs_15f284_10r213.qza --i-classifier gg_13_8_341_805_classifer.qza --o-classification 6.taxonomy_15f284_10r213.qza --p-n-jobs 64

#File format qza to qzv

qiime metadata tabulate --m-input-file 6.taxonomy_15f284_10r213.qza --o-visualization 6.taxonomy_15f284_10r213.qzv

# Visualize taxonomy with an interactive bar plot

qiime taxa barplot --i-table 2.table_15f284_10r213.qza --i-taxonomy 6.taxonomy_15f284_10r213.qza --m-metadata-file metadata.txt --o-visualization taxonomy 6.taxonomy_barplot_15f284_10r213.qzv

#ASV table for LEfSe analysis

qiime taxa collapse --i-table 3.table_15f284_10r213_minfreq20_minsample3.qza --o-collapsed-table 8.collapse_table.qza --p-level 6 --i-taxonomy 6.taxonomy_15f284_10r213.qza

qiime feature-table relative-frequency --i-table 8.collapse_table.qza --o-relative-frequency-table 9.collapse_table_frequency.qza --output-dir collapse.frequency/

qiime tools export --input-path 9.collapse_table_frequency.qza --output-path collapse.frequency/

biom convert -i collapse.frequency/feature-table_level6.biom -o collapse.frequency/feature-table_level6.txt --header-key "taxonomy" --to-tsv

**RNA-seq analysis using metatranscriptome data**

#Obtaining Salmon using snakemake

$ conda install -n base -c conda-forge mamba

$ conda activate base

$ mamba create -c conda-forge -c bioconda -n snakemake snakemake

$ conda activate snakemake

#made snakefile for salmon

SAMPLES = ["C3", "C5", "E3", "E5"]

rule all:

input:

expand("raw/{sample}_1.fastq.gz", sample=SAMPLES),

expand("raw/{sample}_2.fastq.gz", sample=SAMPLES),

expand("qc/fastqc/{sample}_1.before.trim_fastqc.zip", sample=SAMPLES),

expand("qc/fastqc/{sample}_2.before.trim_fastqc.zip", sample=SAMPLES),

expand("trimmed/{sample}_1.fastq.gz", sample=SAMPLES),

expand("trimmed/{sample}_2.fastq.gz", sample=SAMPLES),

expand("qc/fastqc/{sample}_1.after.trim_fastqc.zip", sample=SAMPLES),

expand("qc/fastqc/{sample}_2.after.trim_fastqc.zip", sample=SAMPLES),

expand("salmon/{sample}/quant.sf", sample=SAMPLES),

expand("salmon/{sample}/lib_format_counts.json", sample=SAMPLES)

rule fastqc_before_trim_1:

input:

"raw/{sample}.fastq.gz"

output:

html="qc/fastqc/{sample}.before.trim.html",

zip="qc/fastqc/{sample}.before.trim_fastqc.zip"

log:

"logs/fastqc/{sample}.before.log"

wrapper:

"0.74.0/bio/fastqc"

rule cutadapt:

input:

r1 = "raw/{sample}_1.fastq.gz",

r2 = "raw/{sample}_2.fastq.gz"

output:

fastq1="trimmed/{sample}_1.fastq.gz",

fastq2="trimmed/{sample}_2.fastq.gz",

qc="trimmed/{sample}.qc.txt"

params:

adapters = "-a AGAGCACACGTCTGAACTCCAGTCAC -g AGATCGGAAGAGCACACGT -A AGAGCACACGTCTGAACTCCAGTCAC -G AGATCGGAAGAGCACACGT",

extra = "--minimum-length 1 -q 20"

log:

"logs/cutadapt/{sample}.log"

wrapper:

"0.74.0/bio/cutadapt/pe"

rule fastqc_after_trim_2:

input:

"trimmed/{sample}.fastq.gz"

output:

html="qc/fastqc/{sample}.after.trim.html",

zip="qc/fastqc/{sample}.after.trim_fastqc.zip"

log:

"logs/fastqc/{sample}.after.log"

wrapper:

"0.74.0/bio/fastqc"

rule salmon_index:

input:

"raw/Mus_musculus.GRCm38.cdna.all.fa.gz"

output:

directory("salmon/transcriptome_index")

log:

"logs/salmon/transcriptome_index.log"

params:

# optional parameters

extra=""

wrapper:

"0.74.0/bio/salmon/index"

rule salmon_quant_reads:

input:

# If you have multiple fastq files for a single sample (e.g. technical replicates)

# use a list for r1 and r2.

r1 = "trimmed/{sample}_1.fastq.gz",

r2 = "trimmed/{sample}_2.fastq.gz",

index = "salmon/transcriptome_index"

output:

quant = 'salmon/{sample}/quant.sf',

lib = 'salmon/{sample}/lib_format_counts.json'

log:

'logs/salmon/{sample}.log'

params:

# optional parameters

libtype ="A",

extra="--validateMappings"

wrapper:

"0.74.0/bio/salmon/quant"

# process snakemake

snakemake -n

#for DESeq2 in R

setwd("/home/baejw/Juneyoung")

if (!requireNamespace("BiocManager", quietly = TRUE))

install.packages("BiocManager")

BiocManager::install()

BiocManager::install(c("tximport", "GenomicFeatures"))

BiocManager::install("GenomicFeatures")

library(tximport)

library(GenomicFeatures)

#@ txdb <- makeTxDbFromGFF(file="Mus_musculus.GRCm38.99.gtf")

txdb2 <- makeTxDbFromGFF(file="gencode.vM24.annotation.gff3.gz") #40s

transcripts(txdb2, columns=c("tx_id", "tx_name"))

genes(txdb2)

txdb2

### extract gene_name in '.gff3'

library(rtracklayer)

aa <- import.gff3("gencode.vM24.annotation.gff3.gz") # 25s

gene.DF <- data.frame(aa$gene_name,aa$gene_id)

u.gene.DF<- unique(gene.DF)

head(u.gene.DF)

###

k <- keys(txdb2, keytype = "TXNAME")

tx2gene <- select(txdb2, k, "GENEID", "TXNAME")

head(tx2gene)

## find quant file from Salmon

setwd("/home/baejw/Juneyoung/quant/")

dir <- getwd()

files = paste0(dir, "/",c("C3","C5","E3","E5"),"/quant.sf")

names(files) <- paste0( c("C3","C5","E3","E5"))

file.exists(files)

txi.salmon <- tximport(files, type = "salmon", tx2gene = tx2gene)

str(txi.salmon)

head(txi.salmon$abundance)

head(txi.salmon$counts)

#@ BiocManager::install("DESeq2")

library(DESeq2)

sampleTable <- data.frame(condition = factor(rep(c("C", "E"), each =2)))

rownames(sampleTable) <- colnames(txi.salmon$counts)

dds <- DESeqDataSetFromTximport(txi.salmon, sampleTable, ~condition)

deseqresult <- DESeq(dds)

deseqresult

res <- results(deseqresult)

res

res05 <- results(deseqresult, alpha=0.05)

summary(res05)

res05

res05ordered <- res[order(res05$padj),]

summary(res05ordered)

res05ordered

write.csv(as.data.frame(res05ordered), file = "condition_results_count.csv")

write.csv(as.data.frame(u.gene.DF), file = "gene_name.csv")
